# Supplementary material for: Self-adaptive amorphous CoOxCly electrocatalyst for sustainable chlorine evolution in acidic brine
Source: Nat Commun. 2023 Sep 2;14:5356. doi: 10.1038/s41467-023-41070-7 (PMC10475099; doi:10.1038/s41467-023-41070-7)
Supplement: Supplementary file 1 — Supplementary Information [file 41467_2023_41070_MOESM1_ESM.pdf]

# Supplementary Information

# Self-adaptive amorphous $CoO_xCl_y$ electrocatalyst for sustainable chlorine evolution in acidic brine

Mengjun Xiao[1, ‡], Qianbao Wu[1, ‡], Ruiqi Ku[2], Liujiang Zhou[3], Chang Long[1], Junwu Liang[4,5,*], Andraž Mavrič[6], Lei Li[1], Jing Zhu[7], Matjaz Valant[6], Jiong Li[8], Zhenhua Zeng[9], Chunhua Cui[1,*]

[1]Molecular Electrochemistry Laboratory, Institute of Fundamental and Frontier Sciences, University of Electronic Science and Technology of China, Chengdu, 610054, China.

[2]School of Physics, Harbin Institute of Technology, Harbin 150001, China.

[3]School of Physics, University Electronic Science and Technology of China, Chengdu, 611731, China.

[4]Optoelectronic Information Research Center, School of Physics and Telecommunication Engineering, Yulin Normal University, Yulin, Guangxi, 537000, China.

[5]Center for Applied Mathematics of Guangxi, Yulin Normal University, Yulin, Guangxi, 537000, China.

[6]Materials Research Laboratory, University of Nova Gorica, Vipavska 13, SI-5000 Nova Gorica, Slovenia.

[7]Department of Chemical Physics, School of Chemistry and Materials Science, University of Science and Technology of China, Hefei, 230026, China.

[8]Shanghai Synchrotron Radiation Facility, Shanghai Advanced Research Institute, Chinese Academy of Sciences, Shanghai, 201210 P. R. China.

[9]Davidson School of Chemical Engineering, Purdue University, West Lafayette, Indiana, 47907, United States.

[*]Correspondence to: jwliang@ylu.edu.cn, chunhua.cui@uestc.edu.cn

[‡]These authors contribute equally

**This file concludes:**

Supplementary Figs. 1 to 67

Supplementary Tables 1 to 15

Supplementary Notes 1 to 6

Supplementary References 1 to 21

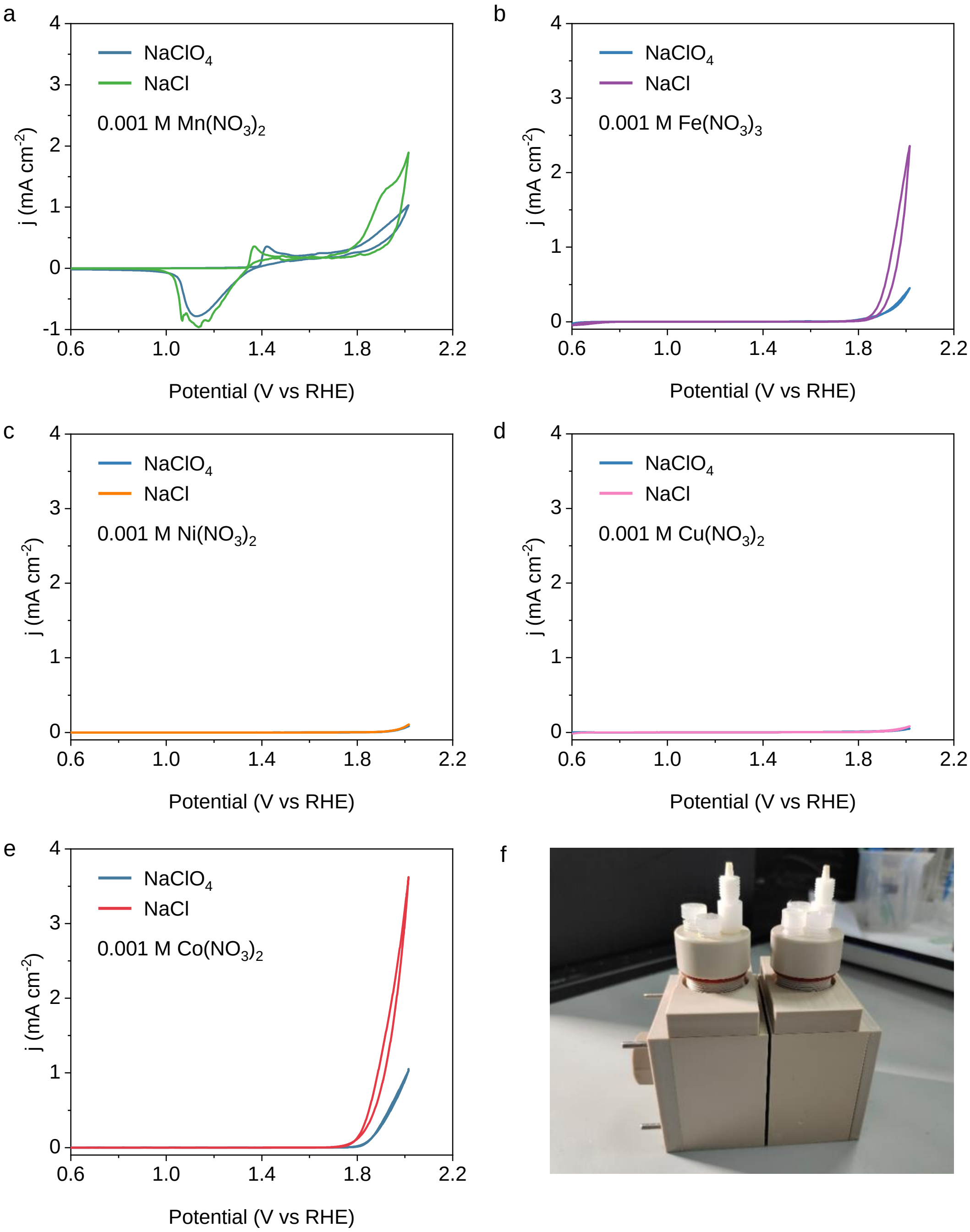


**Supplementary Fig. 1 The CV curves of different metal nitrates for both CER and OER at the first potential cycle at 10 mV s$^{-1}$. (a)** $Mn(NO_3)_2$, **(b)** $Fe(NO_3)_3$, **(c)** $Ni(NO_3)_2$, **(d)** $Cu(NO_3)_2$, and **(e)** $Co(NO_3)_2$. **(f)** A custom-designed PEEK cell for electrolysis. The CER electrolysis was operated in 0.5 M NaCl electrolytes at pH 2 containing 0.001 M metal nitrates. The OER electrolysis was operated in 0.5 M $NaClO_4$ electrolytes at pH 2 containing 0.001 M metal nitrates.

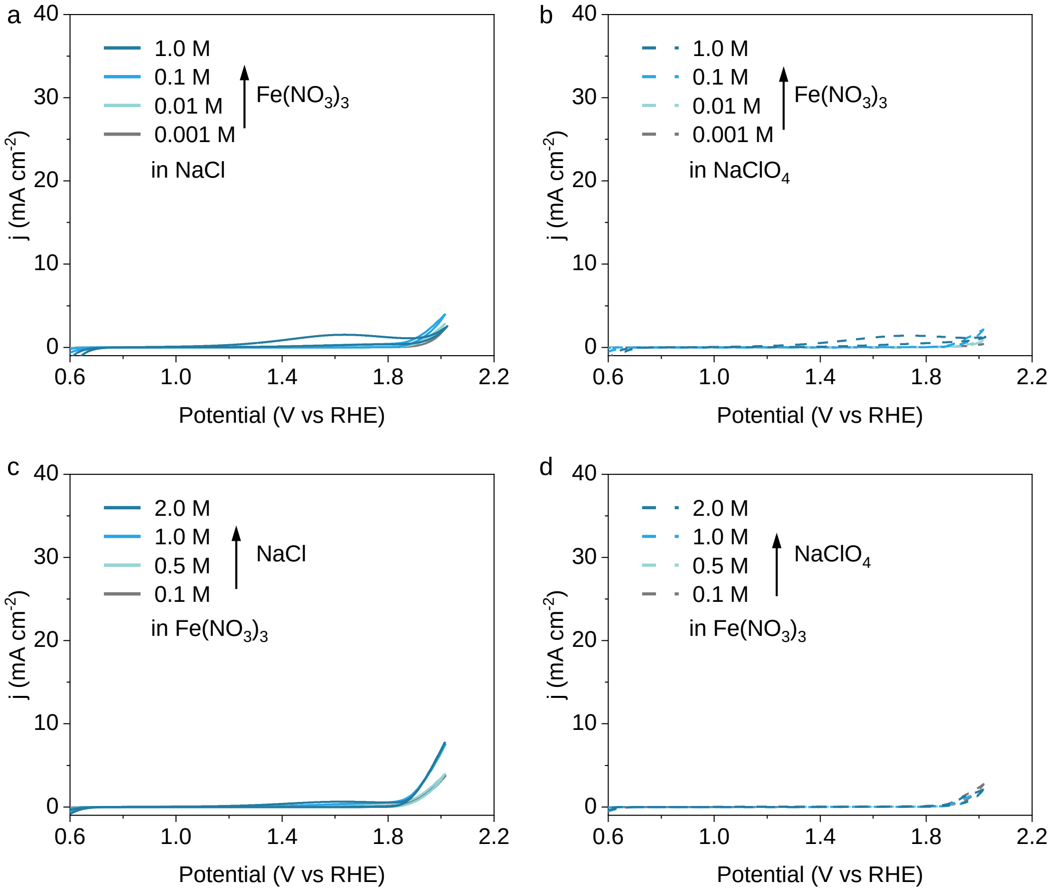


**Supplementary Fig. 2 The CV curves for both CER and OER under different $Fe^{3+}$ and $Cl^-$ concentrations.** The CV curves for CER containing different concentrations of $Fe(NO_3)_3$ in **(a)** 0.5 M NaCl electrolyte and **(b)** 0.5 M $NaClO_4$. The CV curves for CER in 0.1 M $Fe(NO_3)_3$ electrolytes containing different concentrations of **(c)** NaCl and **(d)** $NaClO_4$. All measurements were conducted at pH = 2 and CV curves were recorded at 10 mV $s^{-1}$.

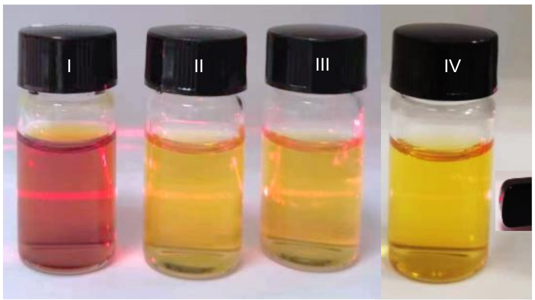


**Supplementary Fig. 3 The hydrolysis of iron.** Color of 1.0 M $Fe(NO_3)_3$ (I), 0.1 M $Fe(NO_3)_3$ (II), 0.01 M $Fe(NO_3)_3$ (III), and 0.001 M $Fe(NO_3)_3$ (IV) solutions in 0.5 M NaCl solution. The light beam showed the hydrolysis of $Fe^{3+}$ for the formation of insoluble colloids.

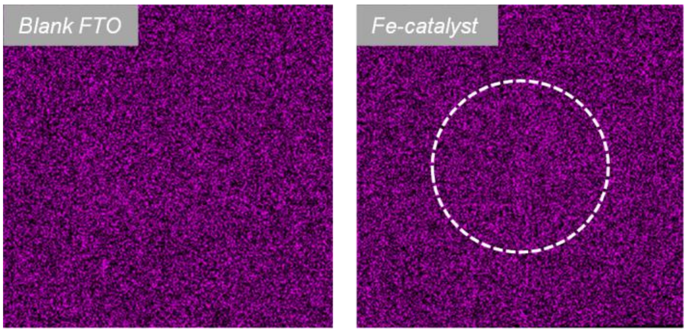


**Supplementary Fig. 4 The X-ray fluorescence (XRF) data.** The XRF Fe Kα mapping of the catalyst film electrodeposited in the electrolyte containing 0.1 M $Fe^{3+}$ and 1.0 M $Cl^-$ at 1.83 $V_{RHE}$ for 2.0 h. XRF mappings showed that there was no significant deposition of Fe.

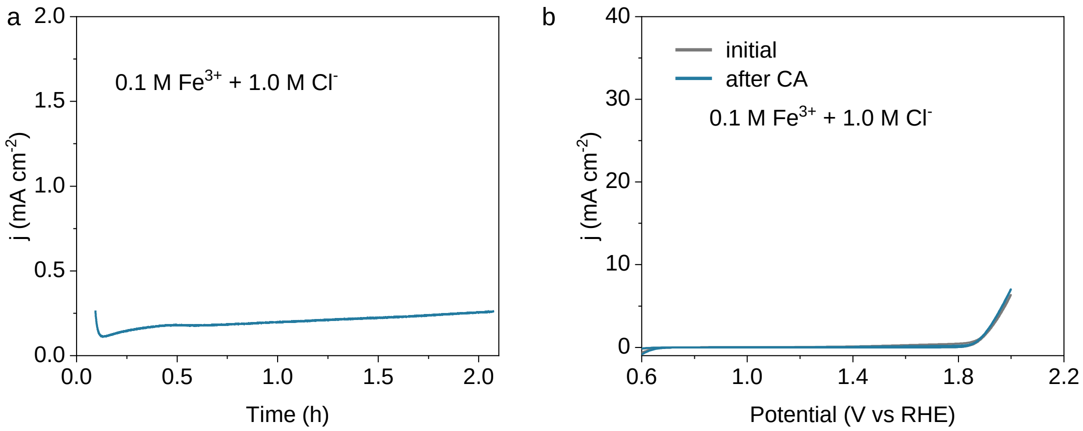


**Supplementary Fig. 5 The CER activity trend with the operating time. (a)** 2 h of electrodeposition operation and **(b)** CV curves of the electrode before and after 2 h of operation at 1.83 $V_{RHE}$ in 0.1 M $Fe(NO_3)_3$ + 1.0 M NaCl electrolyte.

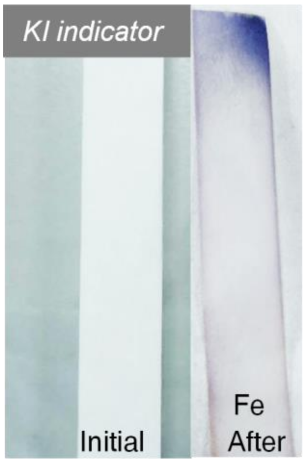


**Supplementary Fig. 6 The determination of $Cl_2$ generation by KI test papers during the LSV.** The wet potassium iodide-starch test paper was positioned in the headspace of a reaction cell during the anodic polarization process. The color change from white to blue-purple suggested the oxidation of $I^-$ to $I_2$ by the electrochemically generated $Cl_2$.

## Supplementary Note 1

As shown in Supplementary Fig. 2, the Fe catalyst only demonstrated initial activity for CER in NaCl compared to $NaClO_4$ electrolyte, yet the CER current densities did not significantly increase with increasing either $Fe^{3+}$ or $Cl^-$ concentrations. This is because the continuous electrodeposition of Fe-catalyst film is not favorable.

In contrast to the Co-catalyst, the Fe-catalyst had two limitations. First, we noticed the dominant hydrolysis of Fe ions even at pH 2 (Supplementary Fig. 3), resulting in the decrease of soluble Fe ions. Second, we showed that only a very small amount of Fe could be deposited on the electrode (Supplementary Fig. 4). Thus, no noticeable increase in current density was observed, and the onset potential remained almost unchanged during the 2-hour operation (Supplementary Fig. 5). Due to the limited number of deposited Fe active sites, the generated $Cl_2$ was little (Supplementary Fig. 6).

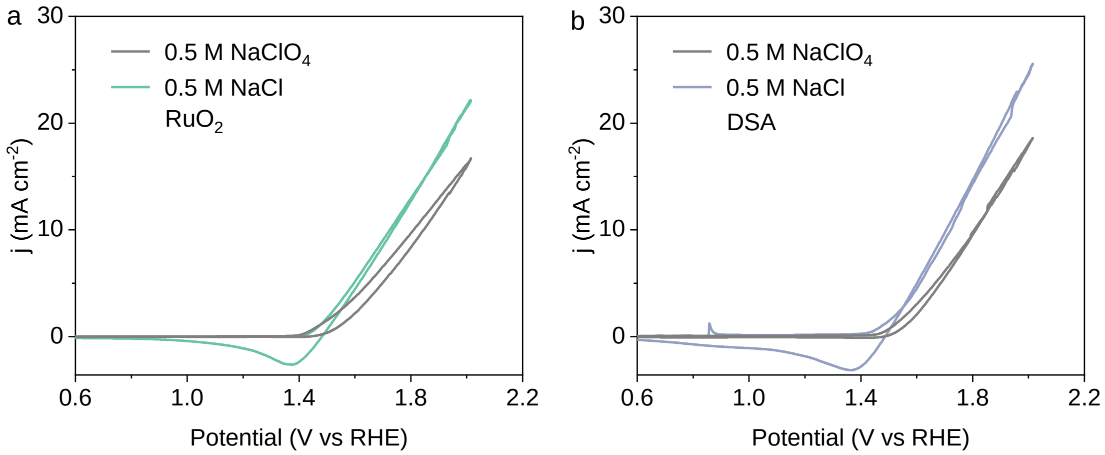


**Supplementary Fig. 7 The CV curves of $RuO_2$ and DSA catalysts for both CER and OER at 10 mV $s^{-1}$. (a)** $RuO_2$ and **(b)** DSA. The CER electrolysis was operated in 0.5 M NaCl electrolytes at pH 2. The OER electrolysis was operated in 0.5 M $NaClO_4$ electrolytes at pH 2.

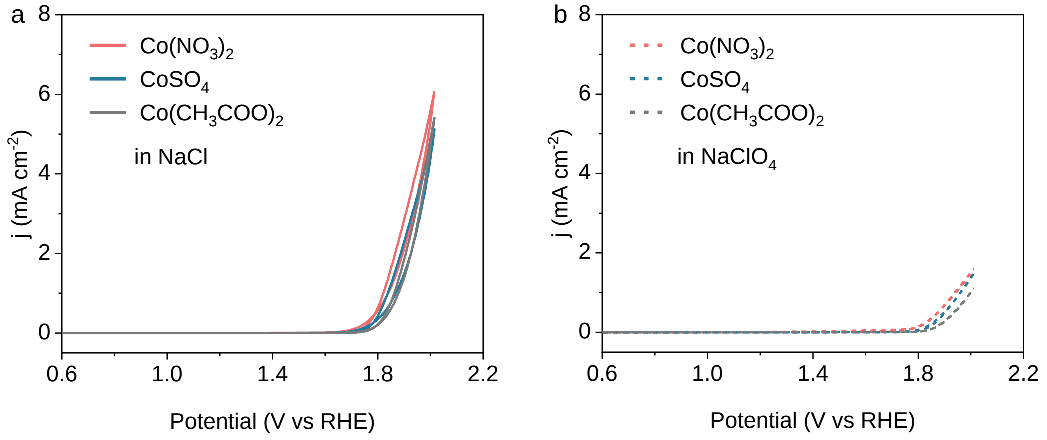


**Supplementary Fig. 8 The CV curves of different Co precursors at the first potential cycle at 10 mV $s^{-1}$.** CV curves of 0.001 M different cobalt precursors for **(a)** CER in 0.5 M NaCl and **(b)** OER in 0.5 M $NaClO_4$ electrolytes at pH = 2. The overlapped CV curves showed that the effect of counter anions of different cobalt salts on both CER and OER is not significant.

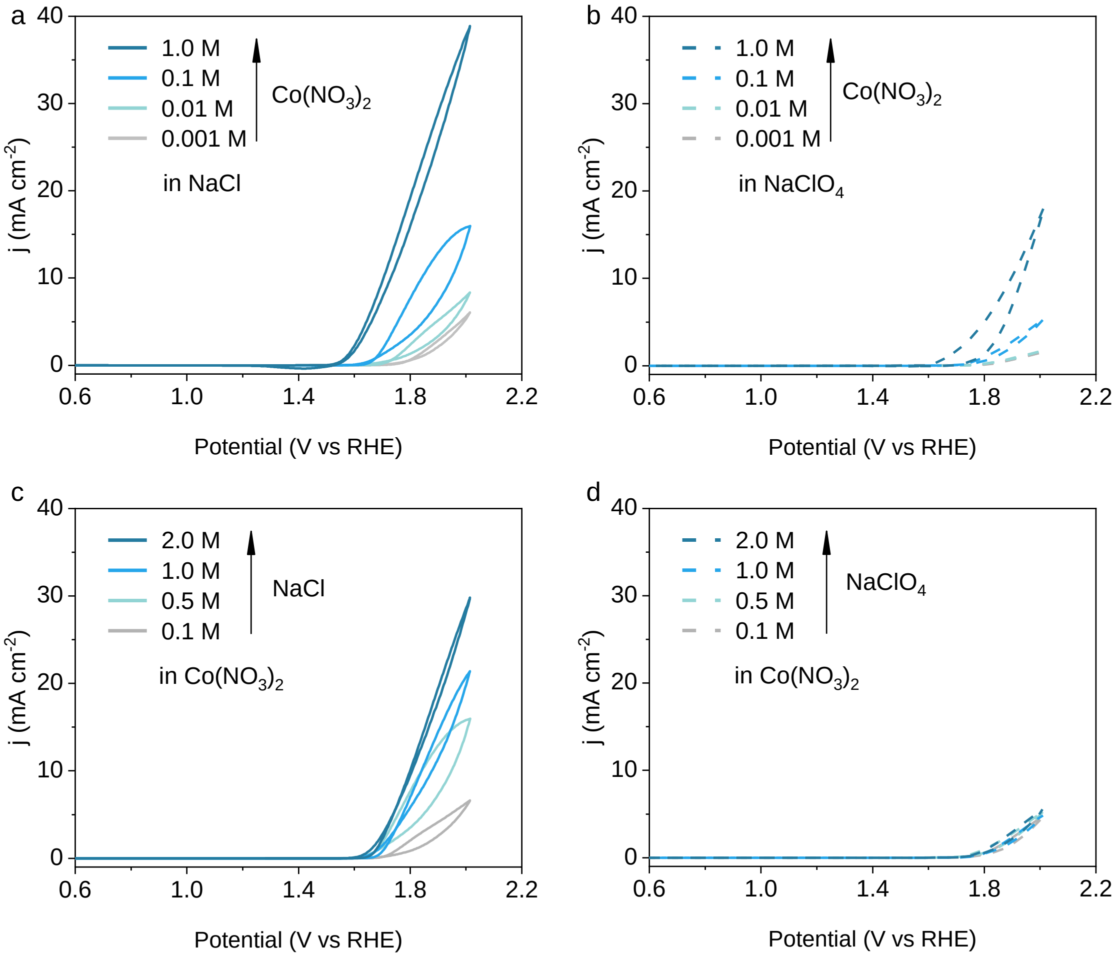


**Supplementary Fig. 9 The CV curves for CER and OER under different $Co^{2+}$ and $Cl^{-}$ concentrations at the first potential cycle. (a)** The CV curves for CER in 0.5 M NaCl electrolyte containing different concentrations of $Co(NO_3)_2$. **(b)** The CV curves for OER in 0.5 M $NaClO_4$ electrolyte containing different concentrations of $Co(NO_3)_2$. **(c)** The CV curves for CER in 0.1 M $Co(NO_3)_2$ electrolytes containing different concentrations of NaCl. **(d)** The CV curves for CER in 0.1 M $Co(NO_3)_2$ electrolytes containing different concentrations of $NaClO_4$. All measurements were conducted at pH = 2 and CV curves were recorded at 10 mV $s^{-1}$.

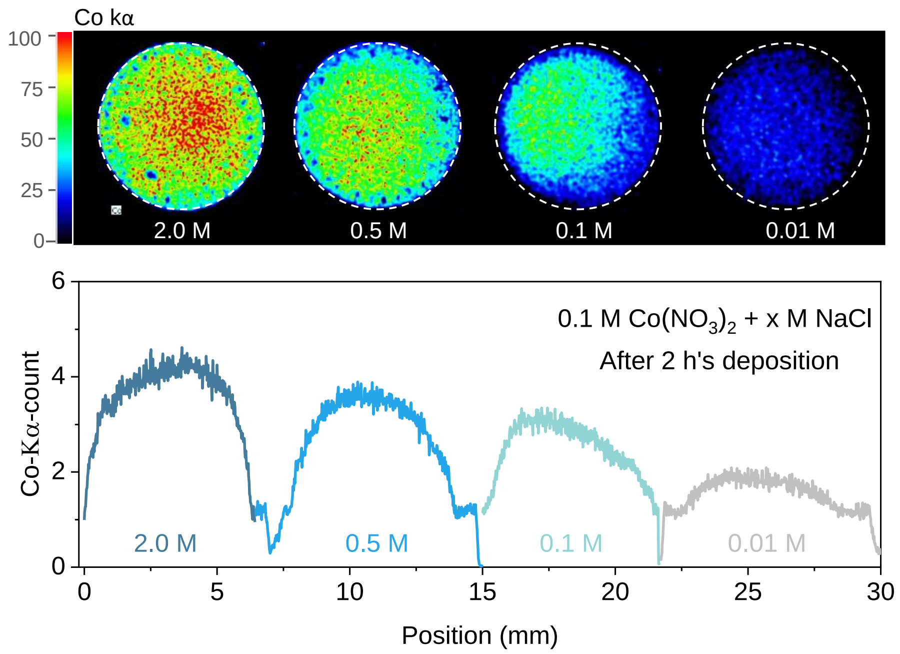


**Supplementary Fig. 10 The Co content of $CoO_xCl_y$ catalyst electrodeposited with different $Cl^-$ concentrations.** (Top) Co Kα X-Ray fluorescence (XRF) mapping image and (bottom) the count of Co Kα for the $CoO_xCl_y$ catalyst after 2 h electrodeposition at 1.67 V with the NaCl concentration from 0.01 to 2.0 M.

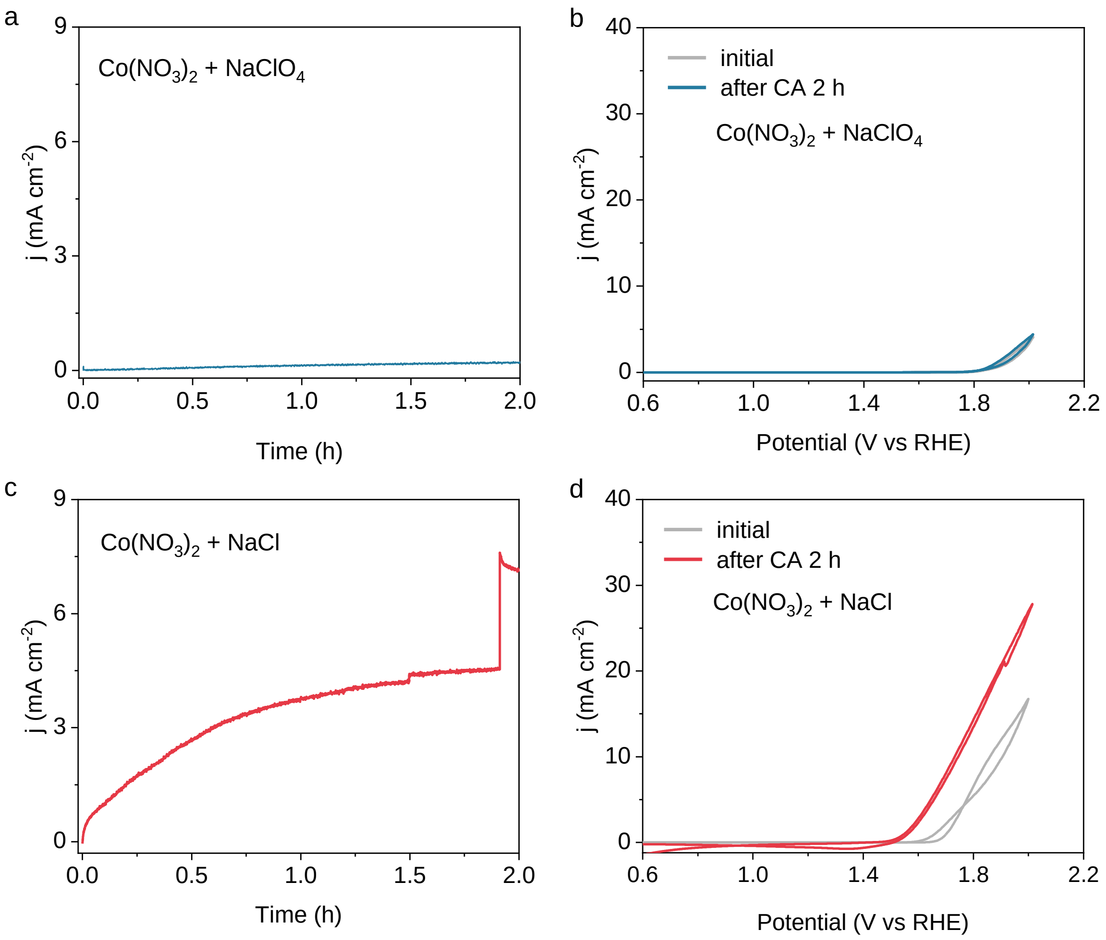


**Supplementary Fig. 11 The influence of electrolyte anions on in situ catalyst electrodeposition and current density. (a)** 2 h of potentiostatic operation and **(b)** CV curves of the electrode before and after 2 h potentiostatic operation at 1.67 V in 0.1 M $Co(NO_3)_2$ + 0.5 M $NaClO_4$ electrolyte. **(c)** 2 h potentiostatic operation and **(d)** CV curves of the electrode before and after 2 h potentiostatic electrolysis at 1.67 V in 0.1 M $Co(NO_3)_2$ + 0.5 M NaCl electrolyte. The jump in the current density in (c) owing to the desorption of the bubble.

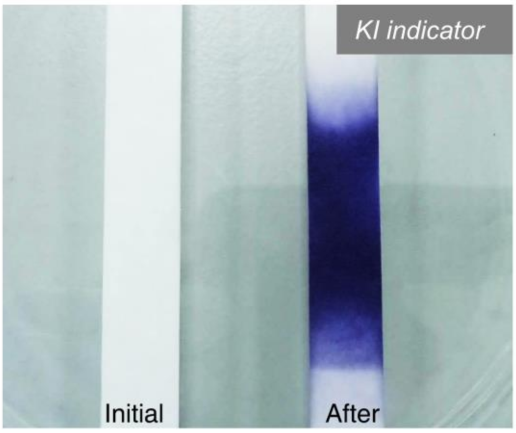


**Supplementary Fig. 12 The determination of $Cl_2$ generation by KI test papers during the LSV.** The wet potassium iodide-starch test paper was positioned in the headspace of a reaction cell during the anodic polarization process. The fast color change from white to blue-purple suggested the oxidation of $I^-$ to $I_2$ by the electrochemically generated $Cl_2$[1].

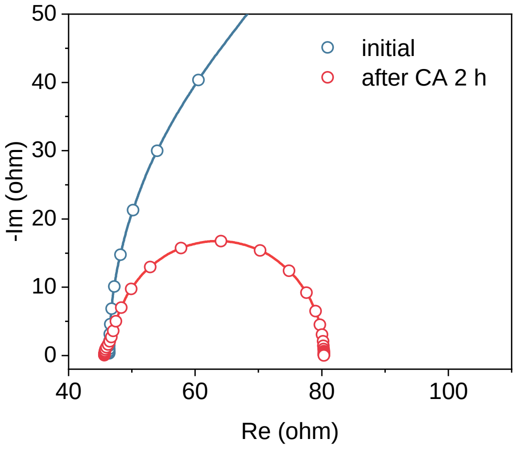


**Supplementary Fig. 13 The impedance spectra.** The impedance of the $CoO_xCl_y$ catalyst before and after 2 h of potentiostatic electrolysis at 1.67 V in 0.1 M $Co^{2+}$ + 0.5 M $Cl^-$ electrolyte.

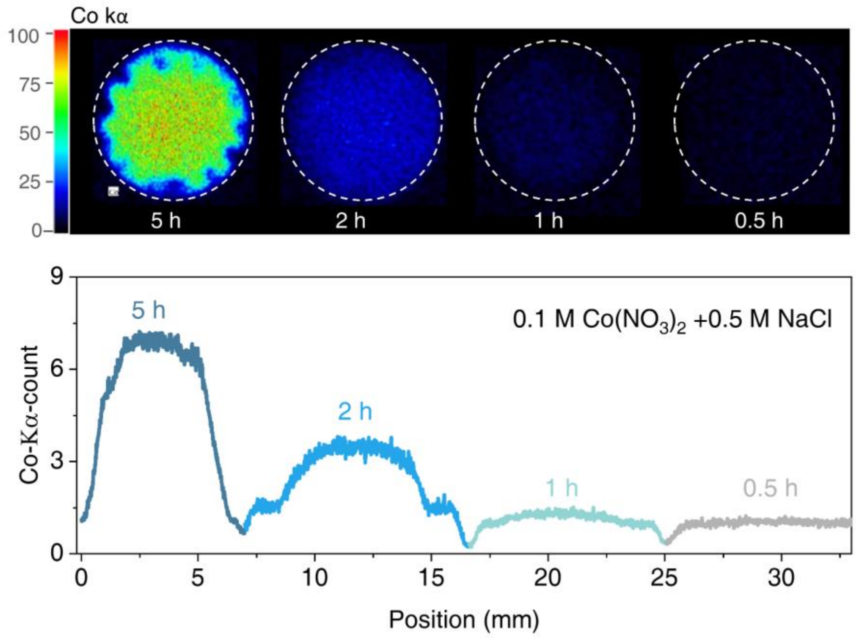


**Supplementary Fig. 14 The Co content test for $CoO_xCl_y$ catalyst with the electrodeposition time.** (Top) Co Kα XRF mapping image and (bottom) the count of Co Kα for $CoO_xCl_y$ catalyst after electrodeposition of an indicated time at 1.67 V.

**Supplementary Note 2 The measurement of the collection efficiency of rotating ring-disk electrode (RRDE).** The collection efficiency was measured based on a high redox reversible ferrocyanide/ferricyanide couple. The experiment was conducted on a freshly prepared blank GC electrode with a Pt ring. 0.1 M Ar-saturated $KNO_3$ aqueous solution containing 0.01 M of $K_3Fe(CN)_6$ was used as the electrolyte. The RRDE was operated with a rotation speed between 400 and 2500 RPM[2-4]. The LSV curves on the disk electrode were recorded at 10 mV $s^{-1}$ at 25 °C via a positive scan mode while the ring electrode potential was fixed at 0.542 V vs Ag/AgCl. Upon the reduction of $Fe(CN)_6^{3-}$ on the disk electrode to $Fe(CN)_6^{4-}$, which can migrate outward to the ring electrode for oxidation, the oxidation current via $Fe(CN)_6^{4-} \rightarrow Fe(CN)_6^{3-} + e^-$ can be recorded simultaneously. The ratio between the ring-limiting current and the disk-limiting current was the collection efficiency, as described by the following equation:

$$N_l = \left| (i_{ring} \times n_D / i_{disk} \times n_R) \right|$$

Where the $i_{ring}$ and $i_{disk}$ are the current density of the ring and disk. The $n_D$ and $n_R$ are the numbers of electrons exchanged at the disk and ring, respectively. In the test potential range with different rotation speeds, the collection efficiency was kept constant at $N_l \approx 0.37$.

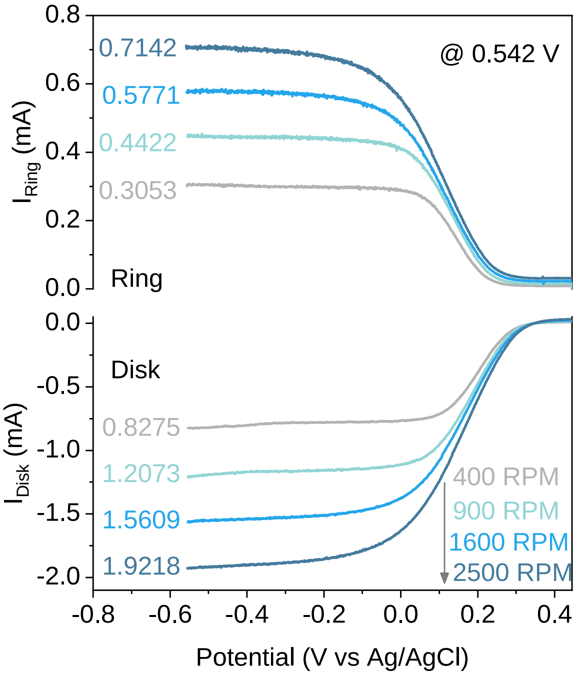


**Supplementary Fig. 15 The RRDE measurement for collection efficiency.** The measurements were conducted in 0.1 M Ar-saturated $KNO_3$ aqueous electrolyte containing 0.01 M of potassium ferricyanide with rotation rates between 400 and 2500 RPM. The polarization curves were recorded at 10 mV $s^{-1}$ from -0.6 to 0.4 $V_{RHE}$ and Pt ring potential was fixed at 0.542 V vs Ag/AgCl. The collection efficiency ($N_l$) was estimated to be ~ 0.37.

**Supplementary Note 3 The $Cl_2$ selectivity quantification by RRDE.** To measure the $Cl_2$ selectivity with RRDE, the ring should be operated at a potential that allows for $Cl_2$ reduction instead of $O_2$ reduction[5]. Thus, the onset potential for both $Cl_2$ and $O_2$ reduction on the Pt ring electrode was determined. Before each measurement, the RRDE was treated successively through physical polishing, electrochemical washing, rinsing with deionized water, and ultrasonically cleaning in ethanol for 15 s. Finally, it was dried in the air for further treatment. Subsequently, the Pt ring (operating at 1600 RPM) was further electropolished in 0.5 M $H_2SO_4$ by 30 CVs between -0.1 V and 1.7 V at 500 mV $s^{-1}$. The LSV curves for oxygen reduction reaction (ORR) and chlorine reduction reaction (CRR) on the Pt ring electrode were recorded at 10 mV $s^{-1}$ at 1600 RPM. The ORR LSV curves on the Pt ring were recorded in 0.5 M $Co^{2+}$ and 0.5 M $NaClO_4$ electrolyte at pH = 2 saturated with $O_2$ gas. Similarly, the CRR LSV curves on the Pt ring electrode were conducted in 0.5 M $Co^{2+}$ and 0.5 M NaCl electrolyte at pH = 2 under an Ar-saturated condition. The disk electrode was polarized at 1.67 V to produce $Cl_2$ gas.

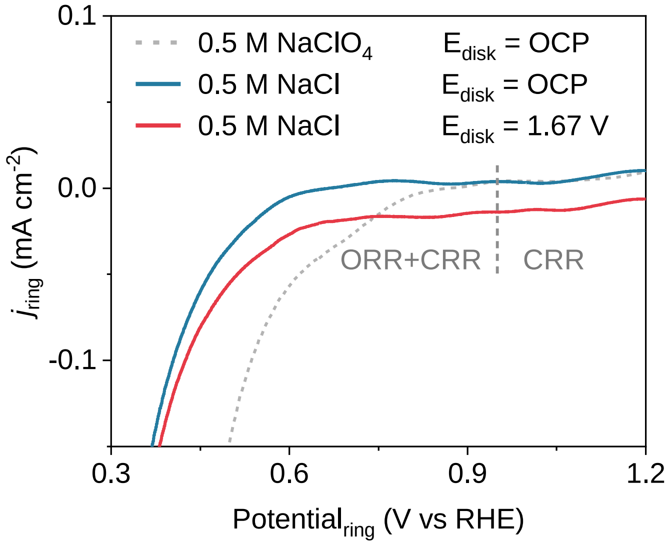


**Supplementary Fig. 16 The $Cl_2$ reduction versus $O_2$ reduction on the Pt ring electrode.** The ring LSV curves were recorded at the forward scan rate of 10 mV $s^{-1}$ at 1600 RPM. The grey dashed curve showed the ring current while the disk was set at the open circuit potential in $Cl^-$ free electrolyte. The blue curve showed the ring current while the disk was set at the open circuit potential in 0.5 M $Cl^-$ electrolyte. The red curve showed the ring current while disk potential was set at 1.67 V in 0.5 M $Cl^-$ electrolyte. The Pt ring potential was fixed at 0.95 V for $Cl_2$ reduction and quantification.

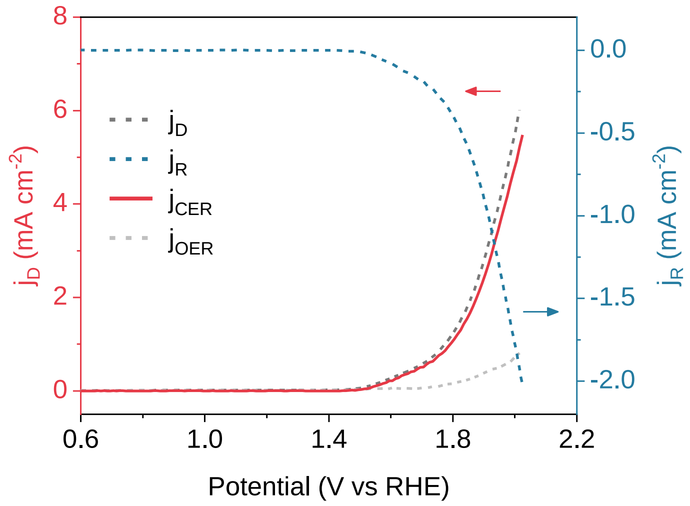


**Supplementary Fig. 17 The determination of $Cl_2$ selectivity at the first potential cycle via RRDE with $CoO_xCl_y$ disk electrode at 1600 RPM**. The $j_D$ and $j_R$ were the respective LSV curves for disk and ring electrodes measured in an Ar-saturated electrolyte containing 0.1 M $Co^{2+}$ and 0.5 M $Cl^-$ at pH 2. The LSV cures were recorded at 10 mV $s^{-1}$. Ring potential was fixed at 0.95 V. The $j_{OER}$ and $j_{CER}$ curves were calculated according to the selectivity of $Cl_2$ determined by the Pt ring electrode (the calculation process was detailed in the Method section).

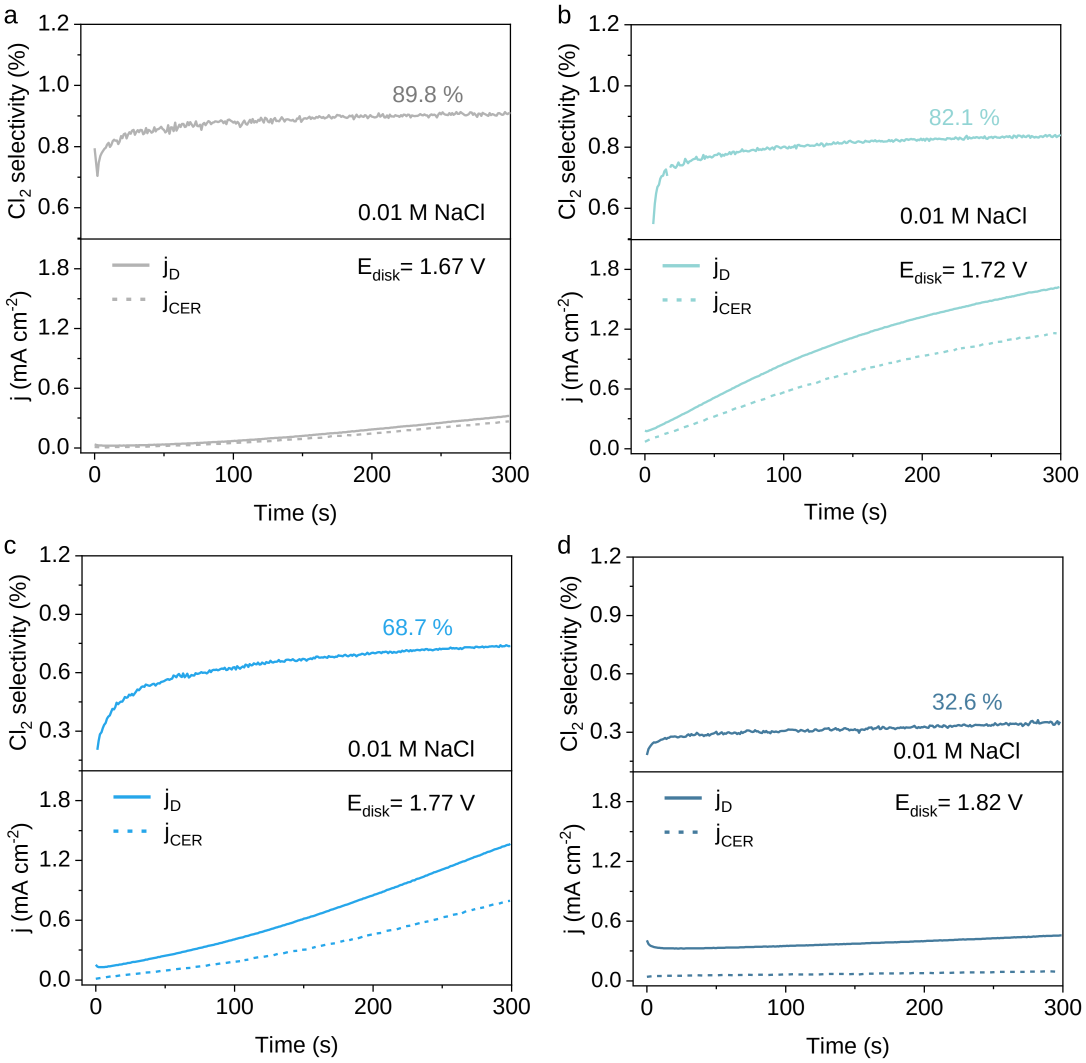


**Supplementary Fig. 18 The determination of $Cl_2$ selectivity via RRDE on $CoO_xCl_y$ catalyst electrode at different potentials in 0.01 M NaCl at pH 2.** The $Cl_2$ selectivity and corresponding i-t curves for the $CoO_xCl_y$ catalyst at **(a)** 1.67 V, **(b)** 1.72 V, **(c)** 1.77 V, and **(d)** 1.82 V. The experiments were conducted in Ar-saturated electrolytes containing 0.1 M $Co^{2+}$ and 0.01 M $Cl^-$ at 1600 RPM.

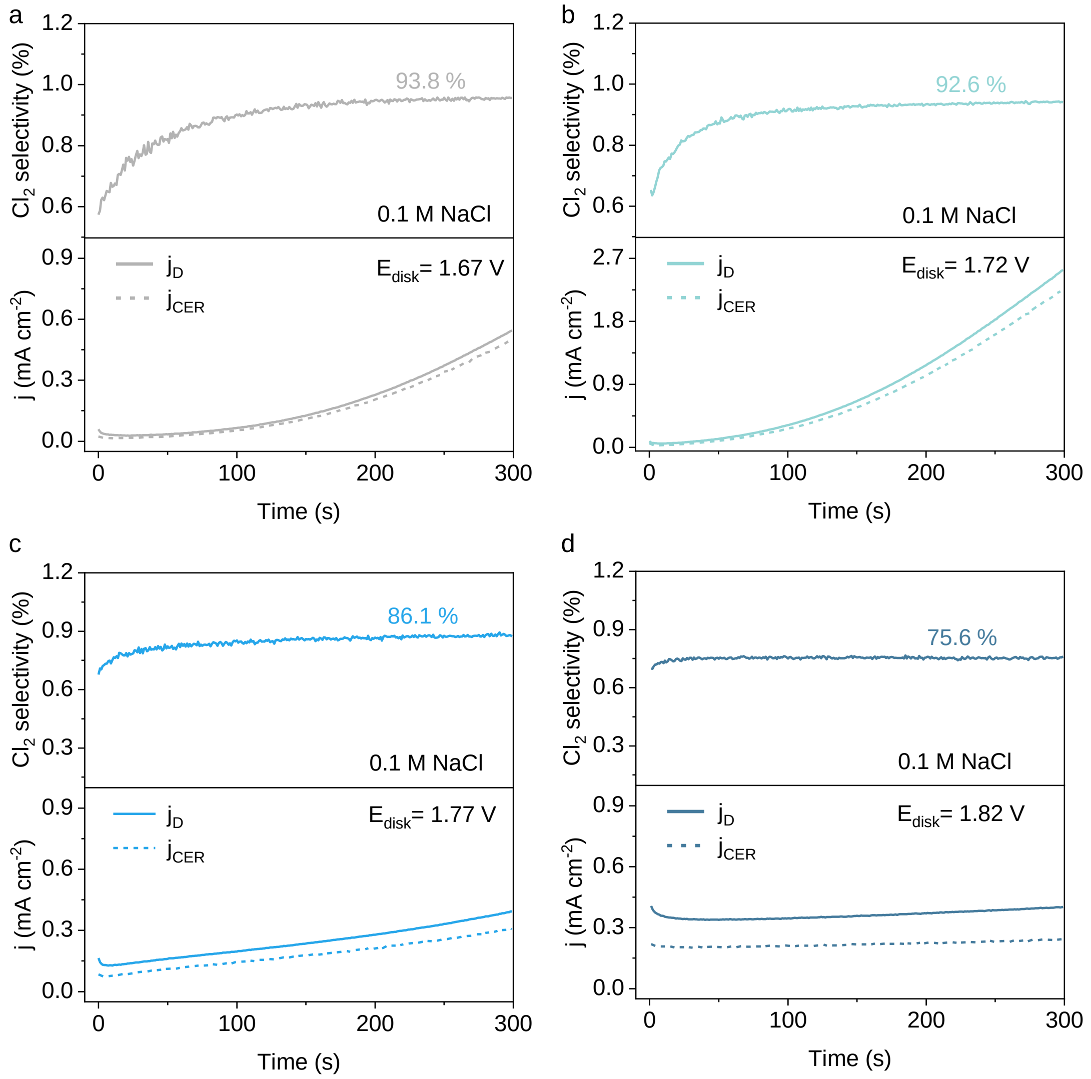


**Supplementary Fig. 19 The determination of $Cl_2$ selectivity via RRDE on $CoO_xCl_y$ catalyst electrode at different potentials in 0.1 M NaCl at pH 2.** The $Cl_2$ selectivity and corresponding i-t curves for the $CoO_xCl_y$ catalyst at **(a)** 1.67 V, **(b)** 1.72 V, **(c)** 1.77 V, and **(d)** 1.82 V. The experiments were conducted in Ar-saturated electrolytes containing 0.1 M $Co^{2+}$ and 0.1 M $Cl^-$ at 1600 RPM.

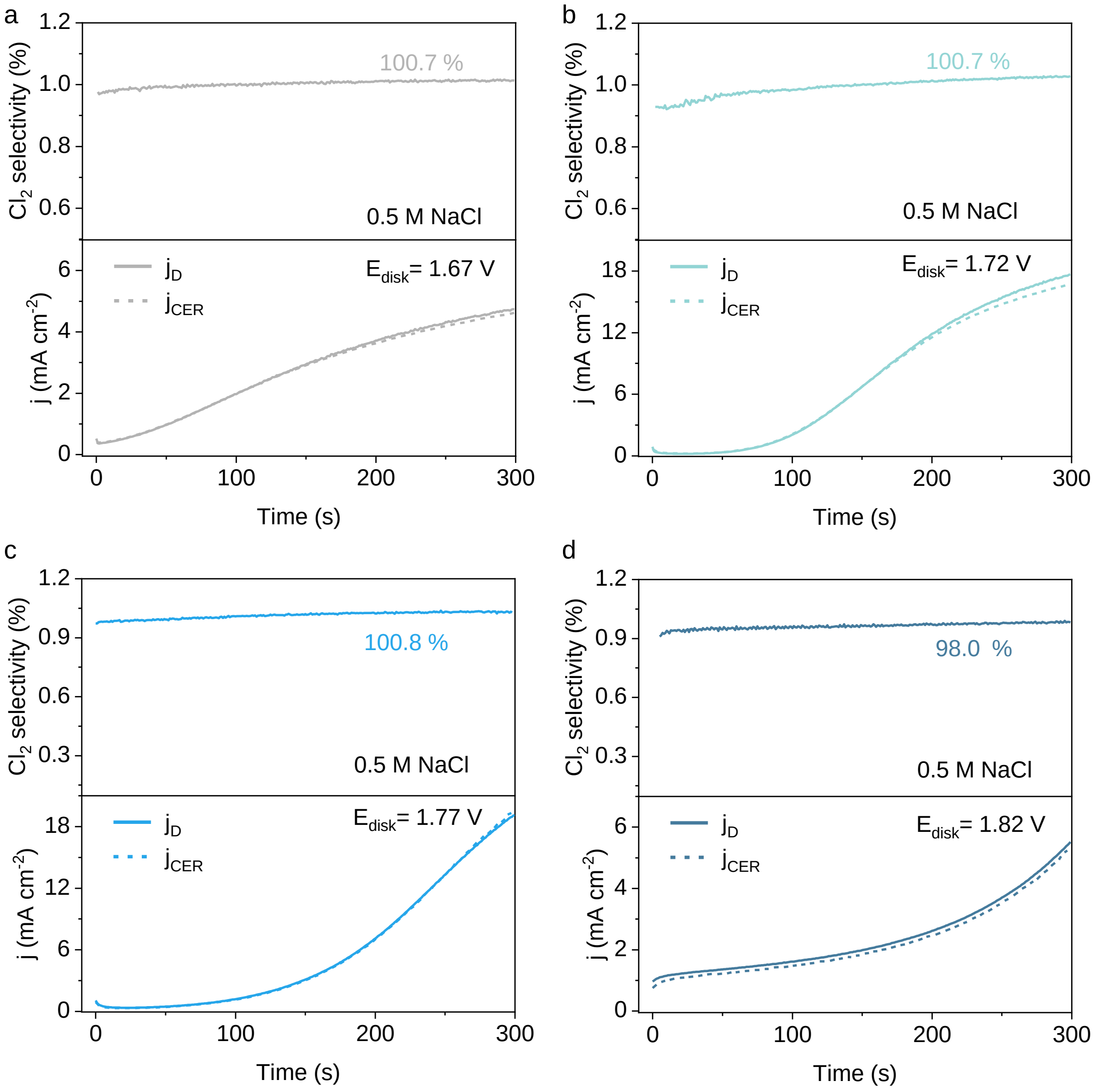


**Supplementary Fig. 20 The determination of $Cl_2$ selectivity via RRDE on $CoO_xCl_y$ catalyst electrode at different potentials in 0.5 M NaCl at pH 2.** The $Cl_2$ selectivity and corresponding i-t curves for the $CoO_xCl_y$ catalyst at **(a)** 1.67 V, **(b)** 1.72 V, **(c)** 1.77 V, and **(d)** 1.82 V. The experiments were conducted in Ar-saturated electrolytes containing 0.1 M $Co^{2+}$ and 0.5 M $Cl^-$ at 1600 RPM.

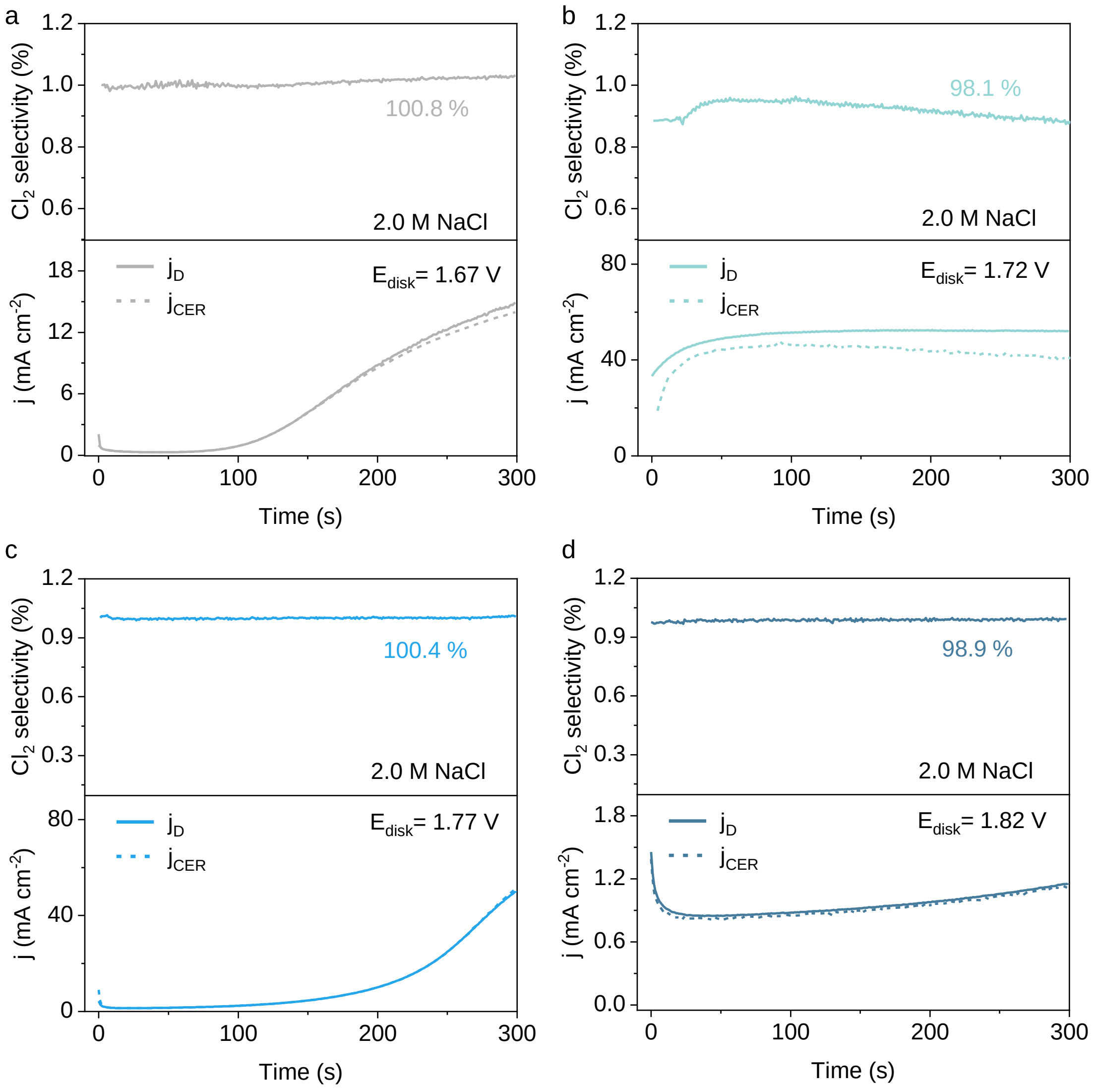


**Supplementary Fig. 21 The determination of $Cl_2$ selectivity via RRDE on $CoO_xCl_y$ catalyst electrode at different potentials in 2.0 M NaCl at pH 2.** The $Cl_2$ selectivity and corresponding i-t curves for the $CoO_xCl_y$ catalyst at **(a)** 1.67 V, **(b)** 1.72 V, **(c)** 1.77 V, and **(d)** 1.82 V. The experiments were conducted in Ar-saturated electrolytes containing 0.1 M $Co^{2+}$ and 2.0 M $Cl^-$ at 1600 RPM.

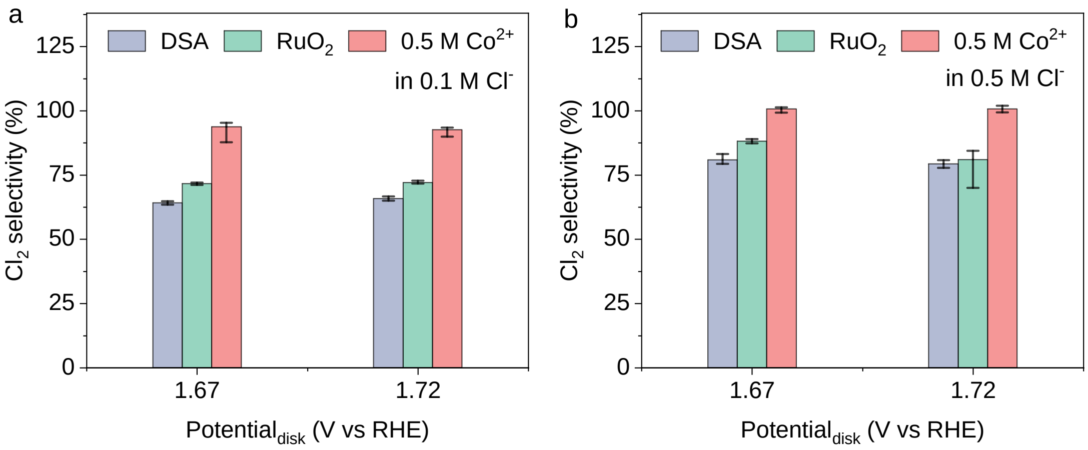


**Supplementary Fig. 22 The CER selectivity for different catalysts.** The $Cl_2$ selectivity of different catalysts under different applied potentials and $Cl^-$ concentrations of **(a)** 0.1 M and **(b)** 0.5 M for the CER process at pH 2. The data were recorded based on a rotating ring disk electrode method at 1600 RPM. The error bars represent the standard deviation for triplicate measurements.

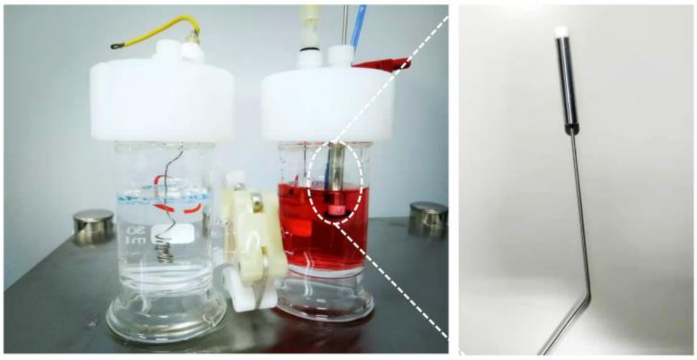

**Supplementary Fig. 23 The photograph of the DEMS experimental device.** For the DEMS measurements, a custom gas-tight electrochemical H-cell with a conventional three-electrode arrangement was employed. In this experiment, a Nafion membrane was used to separate the cathode and anode. The acquisition probe was vertically attached to the working electrode where the volatile species pass through a porous Teflon membrane into the vacuum inlet of the mass spectrometer.

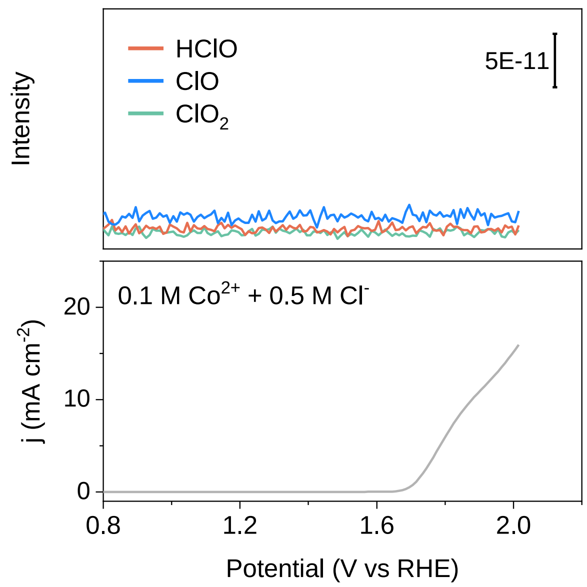


**Supplementary Fig. 24 The LSV curves and corresponding DEMS signals during the CER process at pH 2 at 10 mV $s^{-1}$.** The experiment was carried out in the acidic electrolyte containing 0.1 M $Co^{2+}$ and 0.5 M $Cl^{-}$.

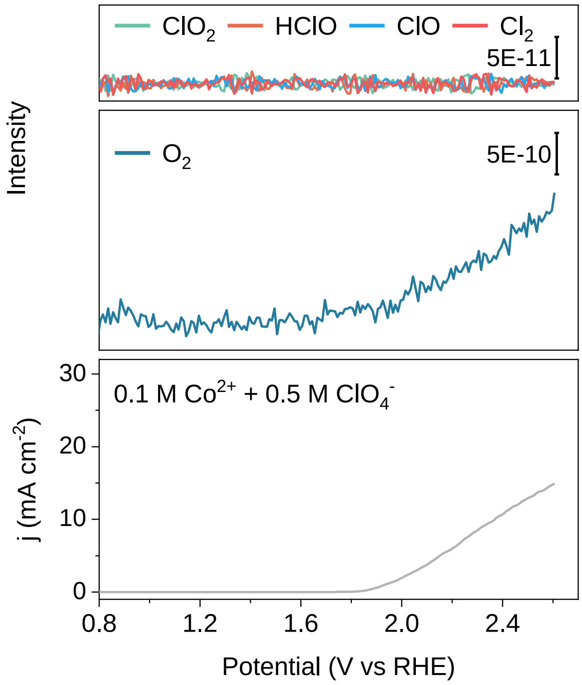


**Supplementary Fig. 25 The LSV curves and corresponding DEMS signals during the OER process at pH 2.** The experiment was carried out in the acidic electrolyte containing 0.1 M $Co^{2+}$ and 0.5 M ClO4$^{-}$.

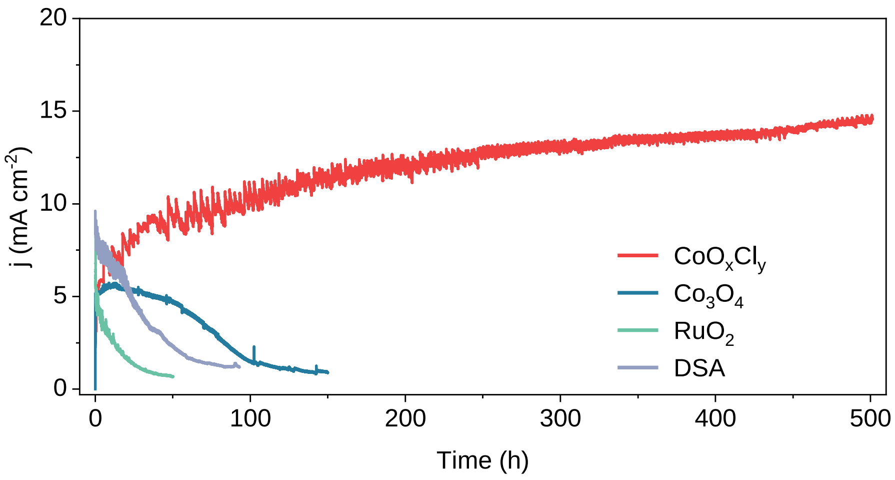


**Supplementary Fig. 26 Long-term stability test of different catalysts electrolysis at 1.67 V in 0.5 M NaCl at pH 2.** This stability test of $CoO_xCl_y$ catalyst starts on a bare FTO without a pre-deposition to present the whole CER trend. For the $CoO_xCl_y$ catalyst, this is a self-adaptive deposition process and the final state of the deposited film depends on a given electrochemical condition, including the applied potential, solution pH, electrolyte component, and concentration. The CER sites were expected to self-optimize to the best stable state. During a long-term process, the introduced Cl in the catalyst film may vary with increasing the thickness of the catalyst film.

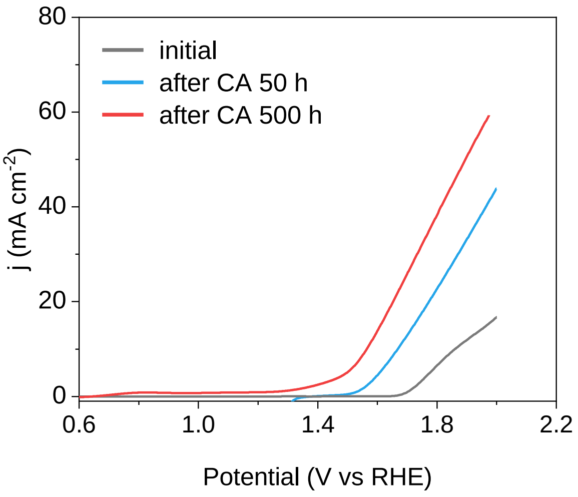


**Supplementary Fig. 27 The LSV curve of $CoO_xCl_y$ catalyst after 50 and 500 h of stability test.** This stability test of the $CoO_xCl_y$ catalyst starts on a bare FTO without a pre-deposition to present the whole CER trend at 1.67 V in the electrolyte containing 0.1 M $Co^{2+}$ and 0.5 M $Cl^-$.

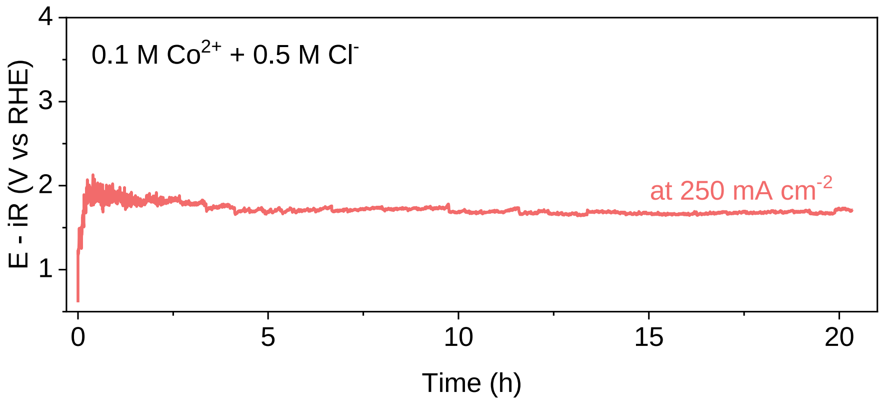


**Supplementary Fig. 28 The stability of $CoO_xCl_y$ catalyst in an acidic saline electrolyte containing $Co^{2+}$ and $Cl^-$ ions at 250 mA $cm^{-2}$.** This stability test of $CoO_xCl_y$ catalyst starts on a bare FTO without a pre-deposition to present the whole CER trend. The potential was iR corrected with 90% ohmic resistance.

**Supplementary Table 1 The performance comparison between different CER catalysts.**

| Catalyst | Electrolyte | Substrate | Selectivity | Stability | Ref. |
|---|---|---|---|---|---|
| $CoO_xCl_y$ | 0.5 M NaCl<br>pH = 2 | FTO | ~100% | ⩾500 h @ 1.67 V<br>⩾20 h @250 mA $cm^{-2}$ | This work |
| $Co_3O_4$ nanobelt | Saturated NaCl<br>pH = 3.1 | FTO | ~ 52% | 10 h @ 1.60 V | 6 |
| $Co_3O_4$ nanoparticles | 0.6 M NaCl<br>pH = 7 | FTO | > 80% | 12 h @10 mA $cm^{-2}$ | 7 |
| Crystalline $CoSb_2O_x$ | 4.0 M NaCl<br>pH = 2.0 | ATO | 97.4± 3.0% | 250 h @100 mA $cm^{-2}$ | 8 |
| $RuO_2NPs@TiO_2$ | 5 M NaCl<br>pH = 3.1 | Ti foil | 90.3% | 12 h @1.53V | 9 |
| $RuO_2@TiO_2$ | saturated NaCl<br>pH = 2 | Ti foil | ~ 90% | 10 h @250 mA $cm^{-2}$ | 10 |
| $IrO_2$-$Ta_2O_5$-$TiO_2$ | 4.0 M NaCl<br>pH = 1 | Ti plate | / | 93 h | 11 |
| $AgCl_2^-$ and $AgCl_3^{2-}$ complex | 1 M NaCl in 0.1 M $HNO_3$ pH = 1 | GC | ~ 71.% | 4 h @1.659 V | 1 |
| Nanoparticles $[Ag_mX_n]^{(m-n)+}$ (X = Cl, Br, I) | 1 M NaCl<br>pH = 1 | GC | ~ 75% | 1.5 h @1. 659 V | 12 |
| $Pt_1$/CNT catalyst | 1.0 M NaCl in 0.1M $HClO_4$ pH=1 | CN | ~ 96.6% | 12 h @ 10 mA $cm^{-2}$ | 13 |

Note: FTO: Fluorine doped tin oxide glass

ATO: Antimony doped tin oxide glass

GC: Glass carbon

CN: Carbon nanotube

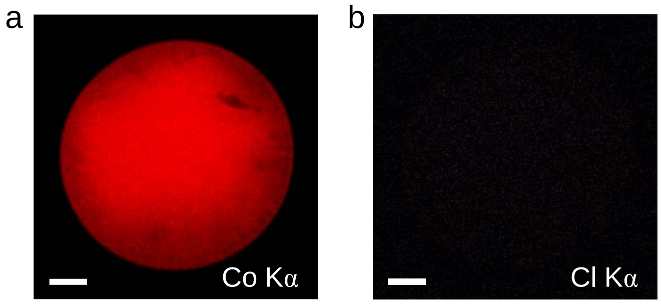


**Supplementary Fig. 29 The XRF mappings of the $CoO_x$ film. (a)** The Co Kα mappings and **(b)** Cl Kα of the $CoO_x$ film. The scale bar is 1 mm. The $CoO_x$ catalyst was electrodeposited at 1.67 V for 10 h in the electrolyte containing 0.1 M $Co^{2+}$ and 0.5 M $ClO_4^-$.

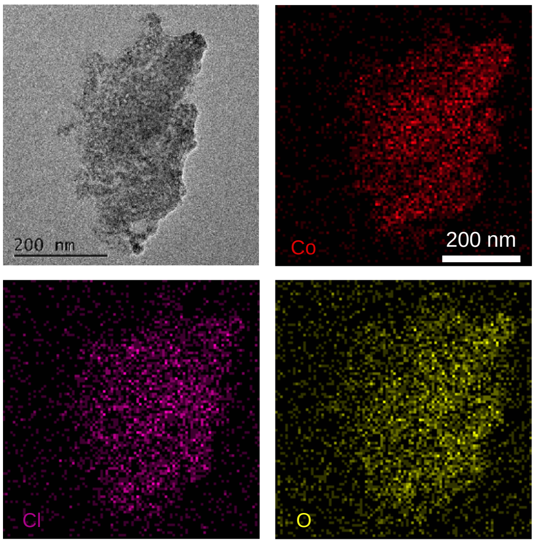


**Supplementary Fig. 30 The TEM image and element mapping of $CoO_xCl_y$ catalyst after 500 h stability test.** The $CoO_xCl_y$ catalyst was electrodeposited at 1.67 V at pH 2 in 0.1 M $Co^{2+}$ + 0.5 M $Cl^-$ electrolyte.

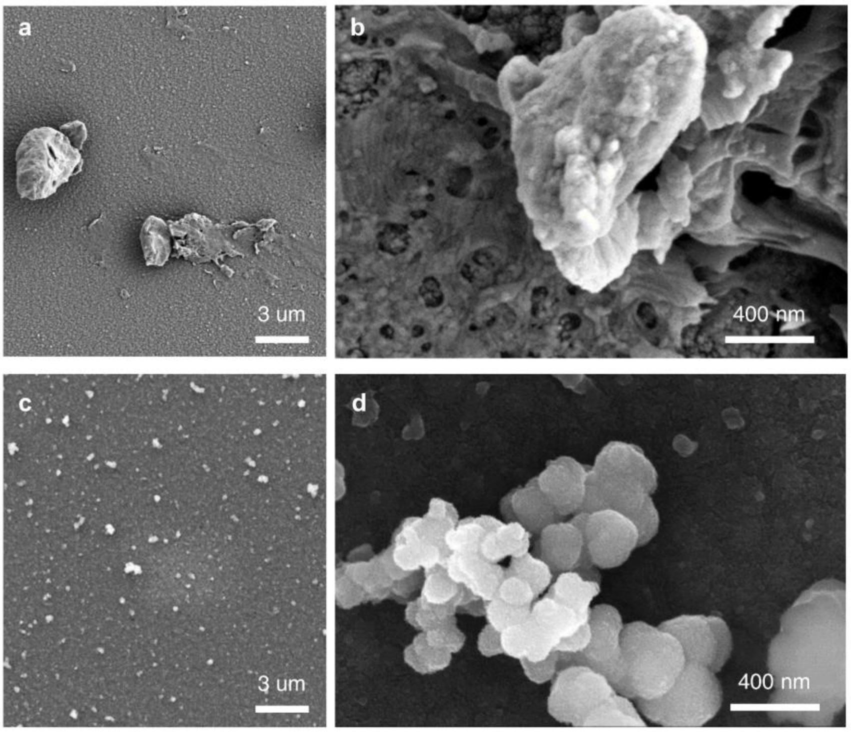


**Supplementary Fig. 31 The SEM images of $CoO_x$ and $CoO_xCl_y$ catalysts. (a)** and **(b)** $CoO_x$ catalysts. **(c)** and **(d)** $CoO_xCl_y$ catalyst. The catalysts were electrodeposited at 1.67 V for 10 h on FTO.

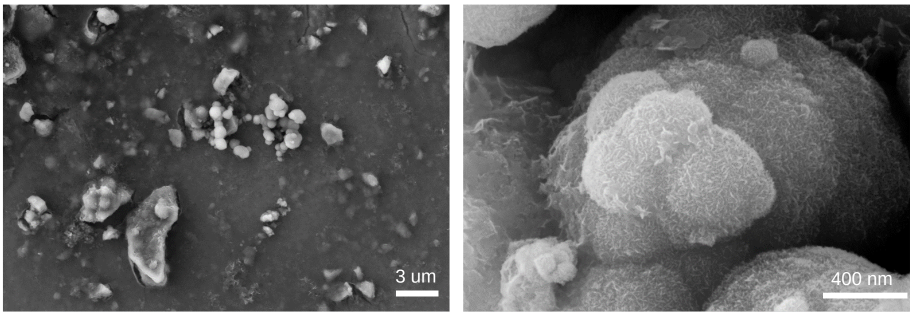


**Supplementary Fig. 32 The SEM images of $CoO_xCl_y$ catalyst.** The $CoO_xCl_y$ film was obtained after the 500 h stability test at 1.67 $V_{RHE}$ in an electrolyte containing 0.1 M $Co^{2+}$ and 0.5 M $Cl^-$.

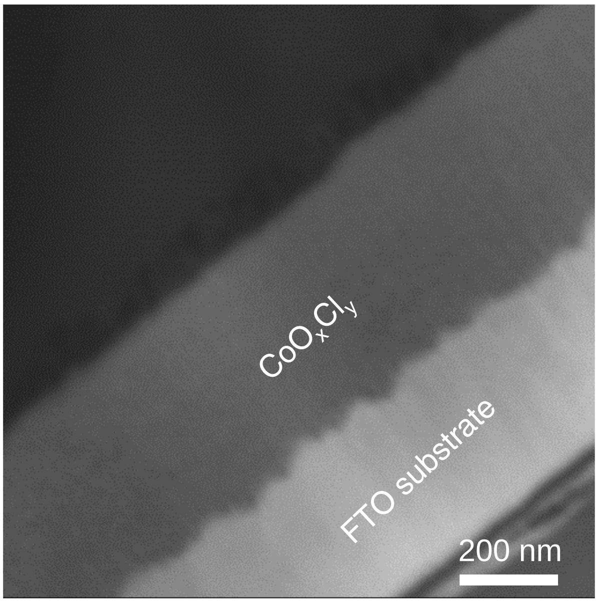


**Supplementary Fig. 33 The SEM images of the cross-section of the $CoO_xCl_y$ catalyst.** The $CoO_xCl_y$ catalyst was electrodeposited at 1.67 V for 40 h at pH 2 in 0.1 M $Co^{2+}$ + 0.5 M $Cl^-$ electrolyte.

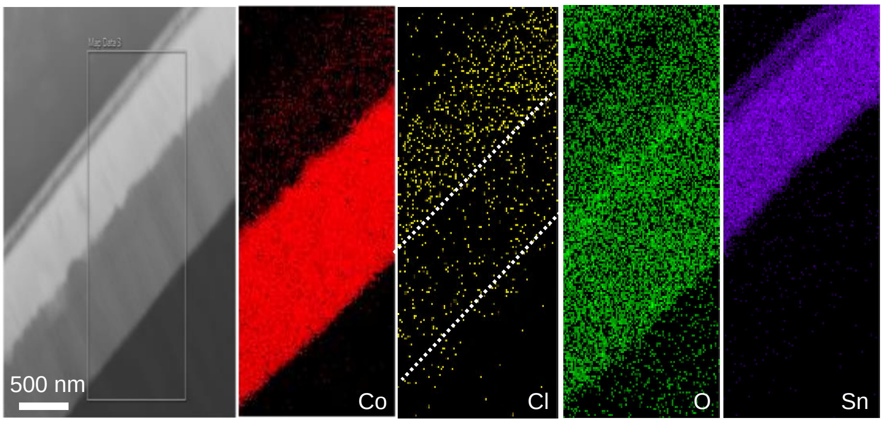


**Supplementary Fig. 34 The EDX mapping of the cross-section of the $CoO_xCl_y$ catalyst.** The $CoO_xCl_y$ catalyst was electrodeposited at 1.67 V for 40 h at pH 2 in 0.1 M $Co^{2+}$ + 0.5 M $Cl^-$ electrolyte.

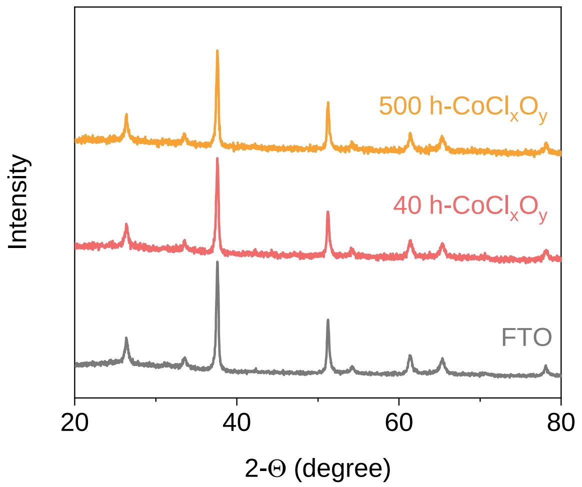


**Supplementary Fig. 35 XRD analysis.** The XRD of the $CoO_xCl_y$ catalysts after 40 h and 500 h of operation at 1.67 $V_{RHE}$ in an electrolyte containing 0.1 M $Co^{2+}$ and 0.5 M $Cl^-$.

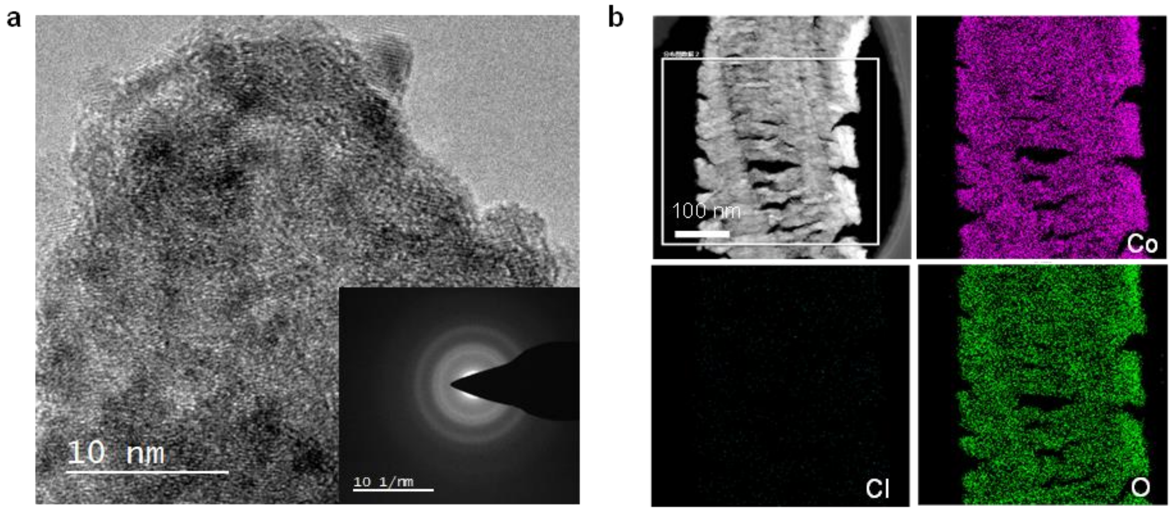


**Supplementary Fig. 36 SEM, SEAD, and DES mapping of $CoO_x$ film. (a)** HRTEM image of $CoO_x$ film electrodeposited at 1.67 V for 40 h and corresponding SAED pattern (inset). **(b)** EDX mappings of $CoO_x$ film. The $CoO_x$ catalyst was electrodeposited at 1.67 V for 40 h at pH 2 in 0.1 M $Co^{2+}$ + 0.5 M $ClO_4^-$ electrolyte.

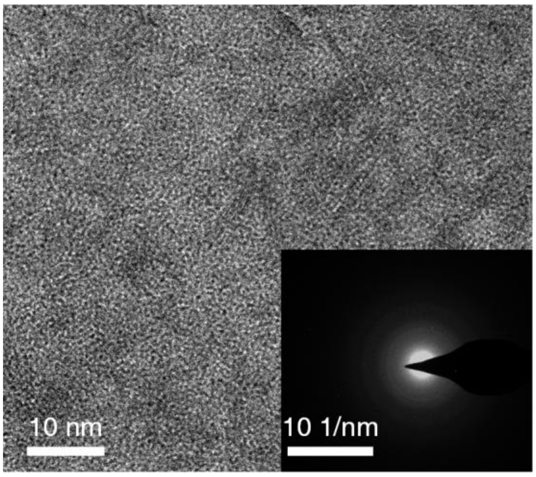


**Supplementary Fig. 37 HRTEM image and the corresponding SAED pattern (inset) of the $CoO_xCl_y$ film after 500 h.** The electrodeposition was conducted at 1.67 $V_{RHE}$ in an electrolyte containing 0.1 M $Co^{2+}$ and 0.5 M $Cl^{-}$.

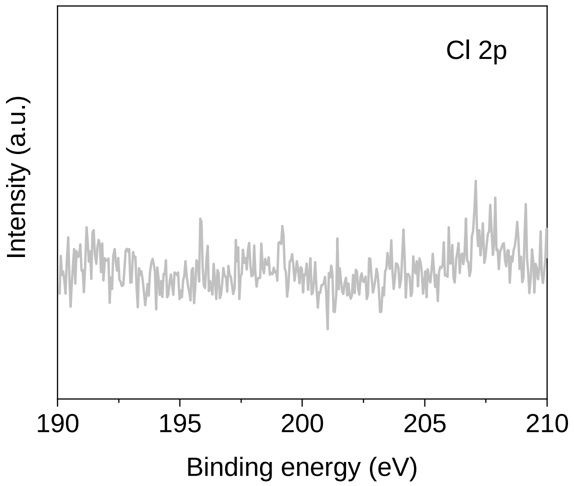


**Supplementary Fig. 38 The Cl 2*p* XPS of $CoO_x$ film.** The $CoO_x$ catalyst was electrodeposited at 1.67 V for 2 h at pH 2 in 0.1 M $Co^{2+}$ + 0.5 M $ClO_4^-$ electrolyte.

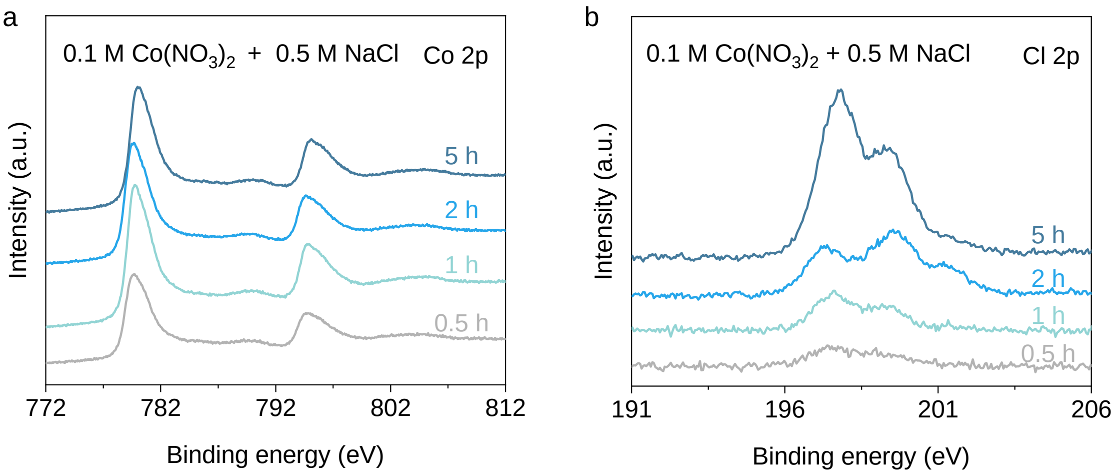


**Supplementary Fig. 39 The XPS spectra of the $CoO_xCl_y$ catalysts prepared through electrodeposition of an indicated time at 1.67 V. (a)** Co 2*p* spectra, and **(b)** Cl 2*p* spectra.

**Supplementary Table 2 The contents of Co, Cl, and O at different deposition times by XPS analysis.**

| Time/h | Co/at % | Cl/at % | O/at % | $CoO_xCl_y$ |
|---|---|---|---|---|
| **0.5** | 11.67 | 1.13 | 43.67 | $CoO_{3.74}Cl_{0.1}$ |
| **1.0** | 12.60 | 1.31 | 41.33 | $CoO_{3.7}Cl_{0.1}$ |
| **2.0** | 13.91 | 3.26 | 42.21 | $CoO_{3.0}Cl_{0.23}$ |
| **5.0** | 15.60 | 6.83 | 43.32 | $CoO_{2.74}Cl_{0.44}$ |

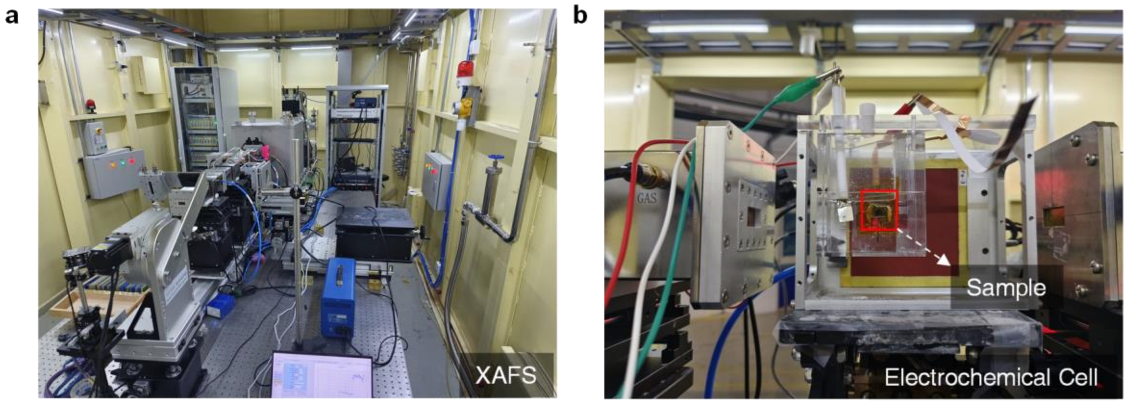


**Supplementary Fig. 40 The images of in situ XAFS. (a)** The in situ XAFS equipment. **(b)** The electrochemical cell.

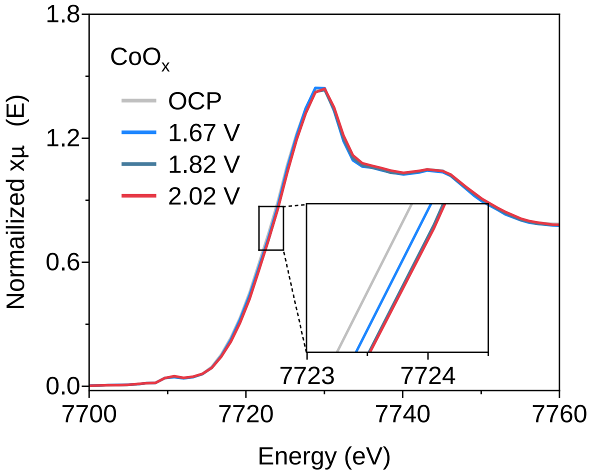


**Supplementary Fig. 41 In situ XAFS measurements.** Co K-edge XANES at different applied potentials from the open-circuit potential to 2.02 V for $CoO_x$ film. The $CoO_x$ films were polarized in electrolytes containing 0.1 M $Co^{2+}$ with 0.5 M $ClO_4^-$.

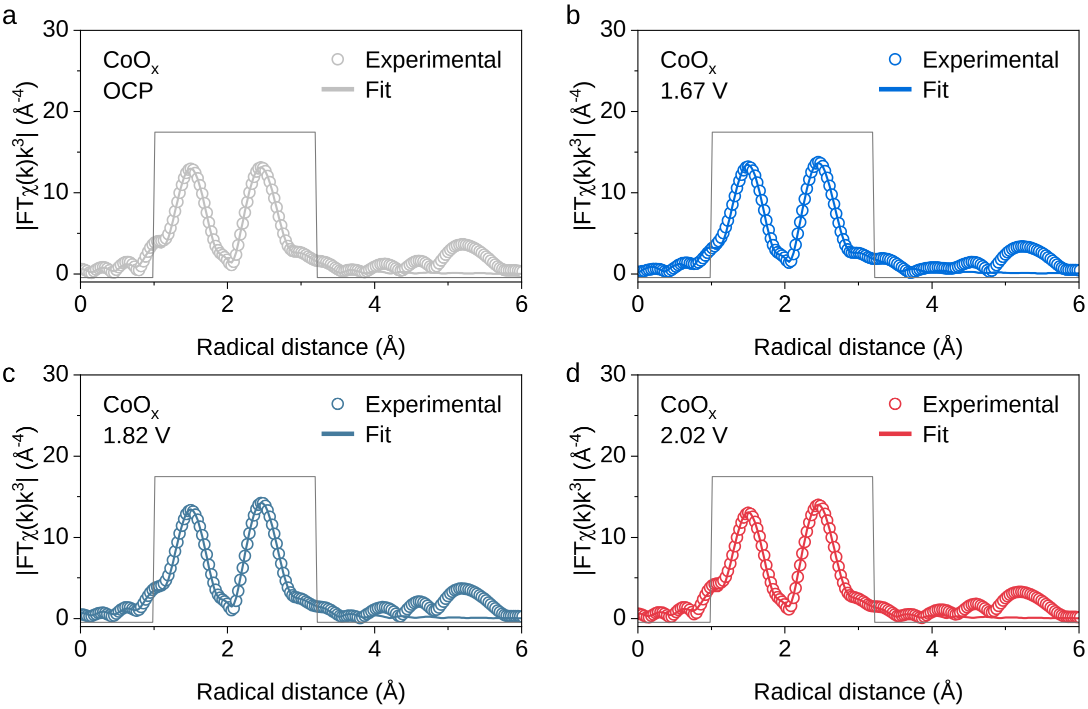


**Supplementary Fig. 42 Fitting curves of Fourier transformed $k^3$-weighted Co K-edge EXAFS in R spaces for $CoO_x$ films.** Fitting curves of Co K-edge EXAFS in R space for $CoO_x$ films at **(a)** OCP, **(b)** 1.67 $V_{RHE}$, **(c)** 1.82 $V_{RHE}$ and **(d)** 2.02 $V_{RHE}$.

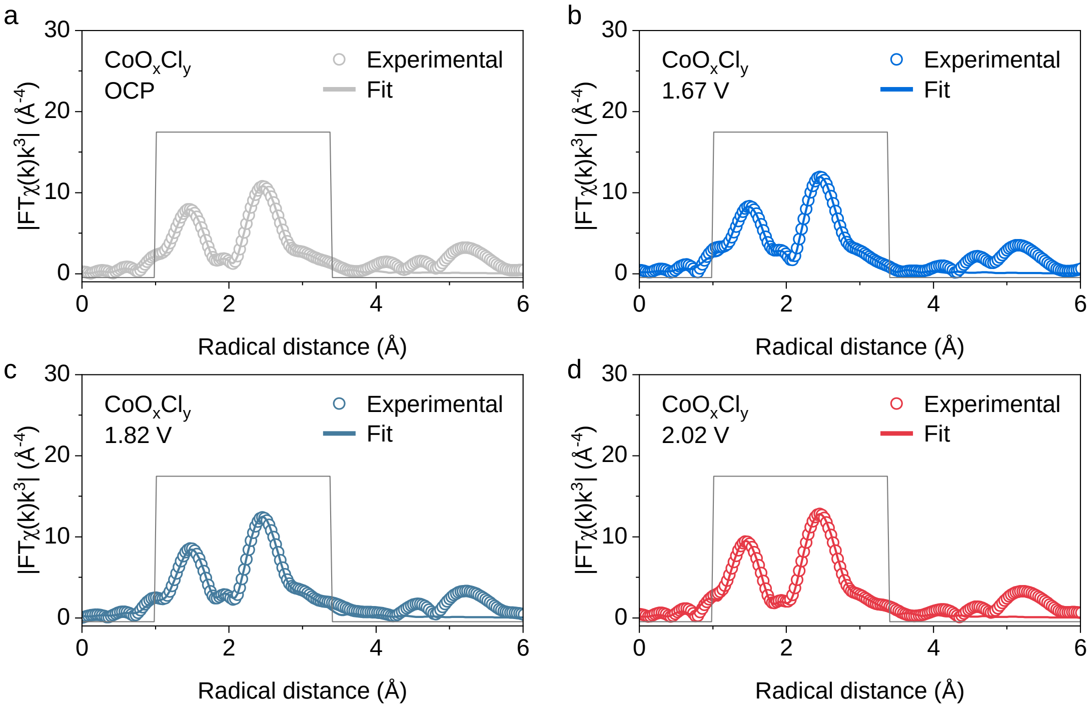


**Supplementary Fig. 43 Fitting curves of Fourier transformed $k^3$-weighted Co K-edge EXAFS in R spaces for $CoO_xCl_y$ films.** Fitting curves of Co K-edge EXAFS in R space for $CoO_xCl_y$ films at **(a)** OCP, **(b)** 1.67 $V_{RHE}$, **(c)** 1.82 $V_{RHE}$, and **(d)** 2.02 $V_{RHE}$.

**Supplementary Table 3 The fitting parameters for FT-EXAFS in R space of $CoO_x$ catalyst.**

| | Path | CN | *R*(Å) | $\sigma^2$(Å$^2$) | $\Delta E_0$(eV) | *R* factor |
|---|---|---|---|---|---|---|
| OCP | Co-O | 5.30±0.17 | 1.903±0.001 | 0.0022±0.0004 | 0.8±0.2 | 0.0014 |
| | Co-Co | 5.42±0.32 | 2.848±0.001 | 0.0047±0.0005 | 0.1±0.3 | |
| | Co-Co | 1.22±0.28 | 3.466±0.001 | | | |
| 1.67 V | Co-O | 5.59±0.21 | 1.901±0.001 | 0.0023±0.0005 | 1.5±0.3 | 0.0016 |
| | Co-Co | 5.61±0.39 | 2.850±0.001 | 0.0047±0.0006 | 1.2±0.3 | |
| | Co-Co | 1.28±0.32 | 3.465±0.001 | | | |
| 1.82 V | Co-O | 5.67±0.18 | 1.902±0.001 | 0.0025±0.0004 | 1.9±0.2 | 0.0014 |
| | Co-Co | 5.74±0.33 | 2.848±0.001 | 0.0046±0.0004 | 1.5±0.3 | |
| | Co-Co | 1.03±0.28 | 3.461±0.001 | | | |
| 2.02 V | Co-O | 5.77±0.21 | 1.902±0.001 | 0.0024±0.0005 | 1.3±0.3 | 0.0019 |
| | Co-Co | 5.88±0.39 | 2.845±0.001 | 0.0045±0.0005 | 0.2±0.3 | |
| | Co-Co | 1.01±0.32 | 3.463±0.001 | | | |

C.N.: Coordination number; R: interatomic distance; $\sigma^2$ : disorder factors; $\Delta E_0$: energy shifts; The amplitude factor $S_0^2$ was determined by fitting standard Co foil with a fixed coordination number based on the known crystal structure.

**Supplementary Table 4 The fitting parameters for FT-EXAFS in R space of $CoO_xCl_y$ catalyst.**

| | Path | C.N. | *R*(Å) | $\sigma^2$(Å$^2$) | Δ$E_0$(eV) | *R* factor |
|---|---|---|---|---|---|---|
| OCP | Co-O | 3.63±0.16 | 1.891±0.001 | 0.0025±0.0005 | -3.0±0.3 | 0.0011 |
| | Co-Cl | 0.45±0.13 | 2.367±0.001 | | | |
| | Co-Co | 3.72±0.19 | 2.850±0.001 | 0.0034±0.0004 | 1.2±0.2 | |
| | Co-Co | 0.97±0.15 | 3.461±0.001 | | | |
| 1.67 V | Co-O | 3.79±0.17 | 1.895±0.001 | 0.0024±0.0005 | -1.1±0.4 | 0.0021 |
| | Co-Cl | 0.52±0.17 | 2.353±0.001 | | | |
| | Co-Co | 3.78±0.23 | 2.842±0.001 | 0.0029±0.0004 | 1.1±0.3 | |
| | Co-Co | 0.91±0.19 | 3.464±0.001 | | | |
| 1.82 V | Co-O | 4.02±0.18 | 1.899±0.001 | 0.0020±0.0005 | -0.6±0.4 | 0.0017 |
| | Co-Cl | 0.68±0.14 | 2.346±0.001 | | | |
| | Co-Co | 4.19±0.26 | 2.844±0.001 | 0.0026±0.0004 | 1.0±0.3 | |
| | Co-Co | 1.14±0.21 | 3.461±0.001 | | | |
| 2.02 V | Co-O | 4.60±0.26 | 1.898±0.001 | 0.0013±0.0006 | -3.0±0.4 | 0.0020 |
| | Co-Cl | 0.74±0.18 | 2.334±0.001 | | | |
| | Co-Co | 5.47±0.35 | 2.843±0.001 | 0.0032±0.0005 | 0.6±0.3 | |
| | Co-Co | 1.07±0.28 | 3.459±0.001 | | | |

C.N.: Coordination number; R: interatomic distance; $\sigma^2$ : disorder factors; Δ$E_0$: energy shifts; The amplitude factor $S_0^2$ was determined by fitting standard Co foil with a fixed coordination number based on the known crystal structure.

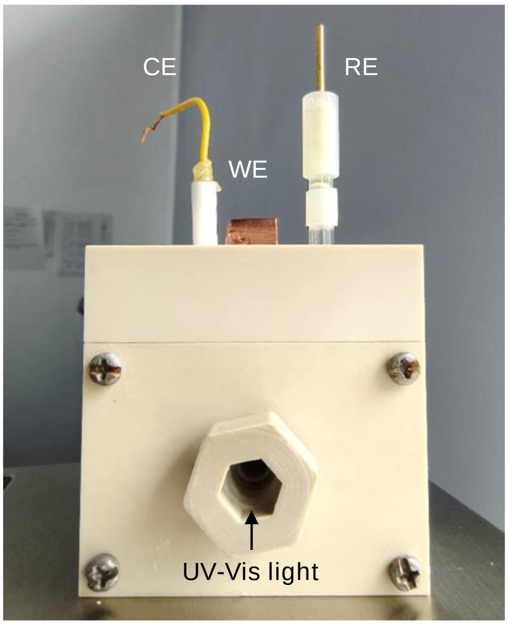


**Supplementary Fig. 44 In situ UV-Vis cell.** For the UV-vis measurements, a custom-designed PEEK cell with a conventional three-electrode arrangement was employed.

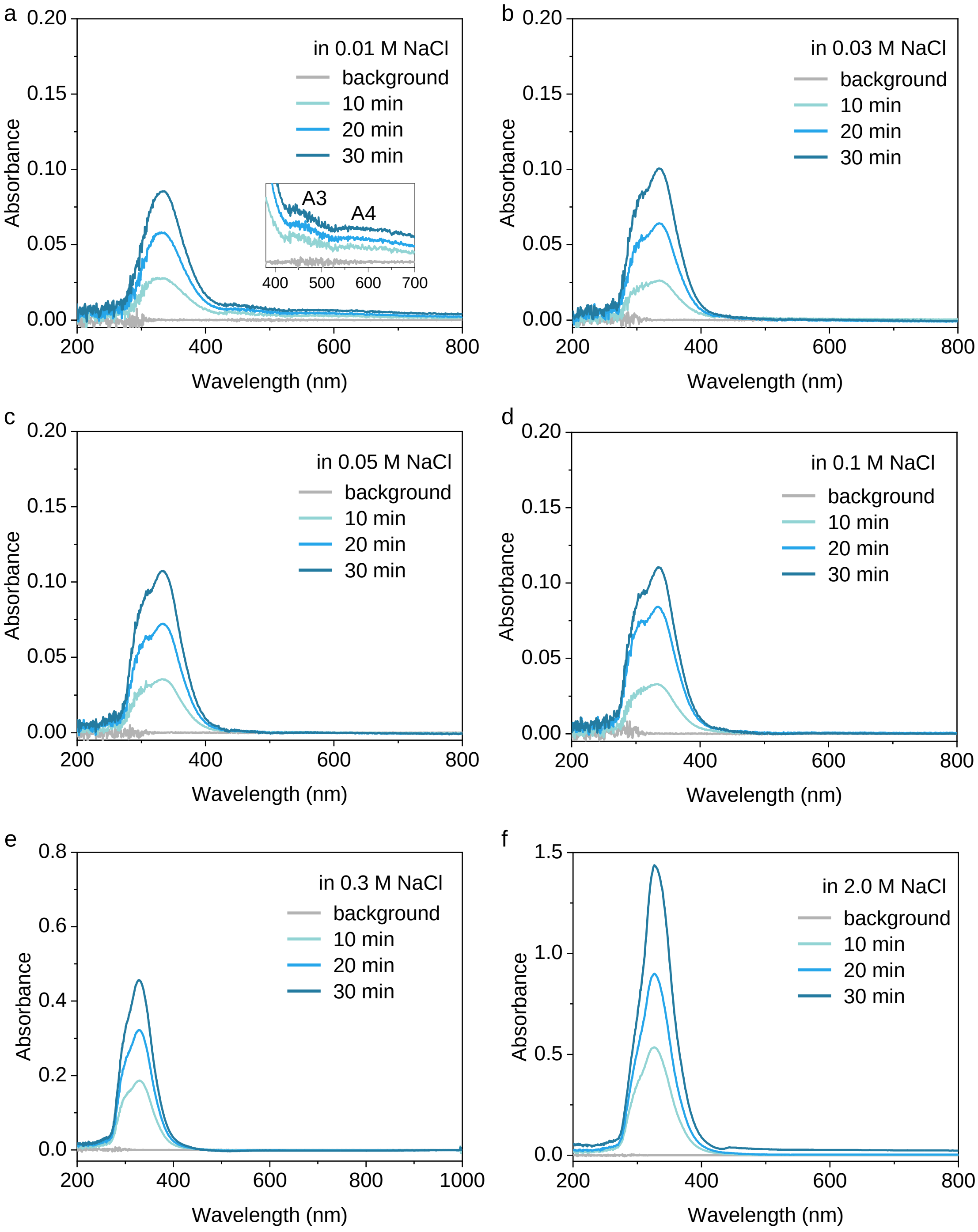


**Supplementary Fig. 45 In situ UV-Vis spectra for tracking the Co coordination states during electrodeposition of $CoO_xCl_y$ film.** The spectra evolution with increasing the deposition time at 1.67 V at pH 2 in 0.1 M $Co(NO_3)_2$ and **(a)** 0.01 NaCl, **(b)** 0.03 M NaCl, **(c)** 0.05 M NaCl, **(d)** 0.1 M NaCl, **(e)** 0.3 M NaCl, and **(f)** 2.0 M NaCl.

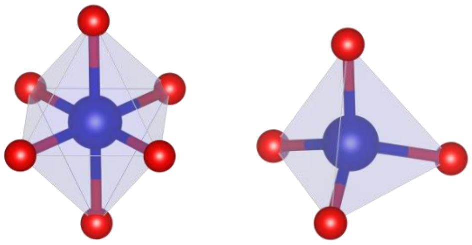

**Supplementary Fig. 46 The octahedral and tetrahedral models for Co oxides.** According to the experiment results, the cobalt oxide model with both octahedral and tetrahedral Co ($Co_{octahedral}$ /$Co_{tetrahedral}$ = 3) was constructed.

**Supplementary Table 5 The charge, magnetic moment ($\mu_B$), and bond length of $Co^{2+}$, $Co^{3+}$, and $Co^{4+}$ used for constructing $Co_3O_4$ and $CoO_2$ calculation models.** The charge is Bader charge. The bond length is the average bond length of Co with oxygen atoms located at vertices of tetrahedral or octahedral.

| **$Co_3O_4$ bulk** | **Charge** | **Magnetic moment** | **Bond length** |
|---|---|---|---|
| **$Co^{2+}$** | 7.63 | 0.07 | 1.94 |
| **$Co^{3+}$** | 7.73 | 2.66 | 1.96 |
| **$CoO_2$ bulk** | **Charge** | **Magnetic moment** | **Bond length** |
| **$Co^{4+}$** | 7.54 | 1.19 | 1.91 |

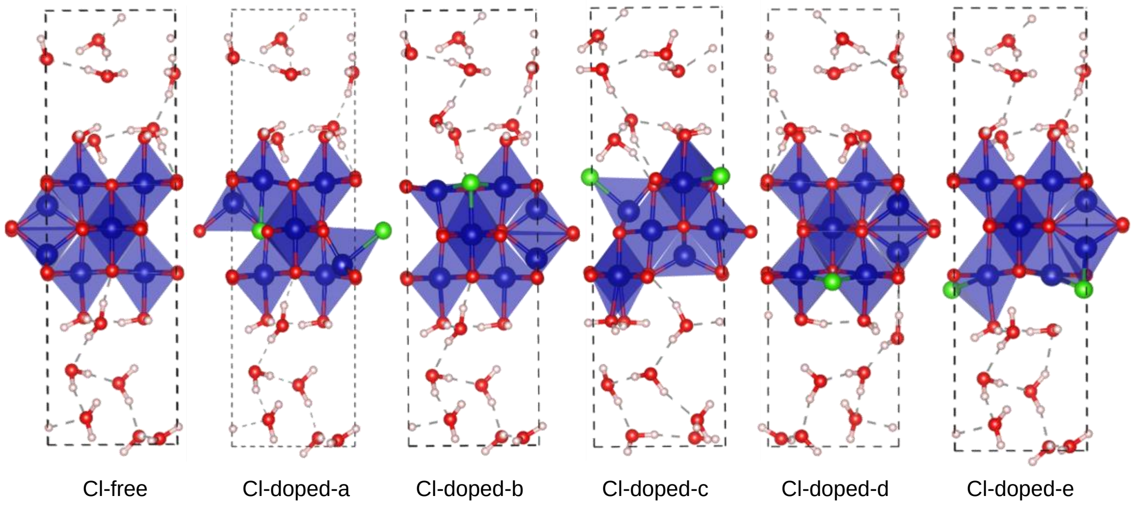


**Supplementary Fig. 47 The optimized structure models for DFT calculation.** According to the experiment results and considering the solvation effect, the hydrous $Co_3O_4$ (100) nanosheet was chosen as the calculation model. On top of the surface, the Co atom located at the octahedral position represents the active site for CER and OER. The blue, red, green, and white balls represent the cobalt, oxygen, chlorine, and hydrogen atom, respectively.

**Supplementary Note 4 The formation energy of models of doping Cl.**

The reaction mechanism of anodic CER usually can be described by the following equations[14]:

$$2Cl^{-}\ (l) \rightarrow Cl_2 + 2e^{-} \qquad (1)$$

The formation energy $dG_f = G(Co_xO_yCl_1) + G(H_2O_{(aq)}) - G(H_{2(g)}) - G(Co_xO_y) - G(Cl^{-} - e^{-})$ (2)

$$E(Cl^{-} - e^{-}) = G(Cl^{-} - e^{-}) - (\Delta ZPE + \Delta H - T\Delta S)_{Cl-} \qquad (3)$$

$$G(Cl^{-} - e^{-}) = 1/2G(Cl_2) - 1.36\ eV \qquad (4)$$

$$\Delta G = \Delta E + \Delta ZPE + \Delta H - T\Delta S \qquad (5)$$

Where ΔE is the change of electronic total energy, ΔZPE is the change in zero-point energy, ΔH refers to the difference in enthalpy of adsorbates, and -TΔS is the entropic correction.

**Supplementary Table 6 Thermodynamic energy correction is used in free energy calculations.** The thermodynamic energy correction ΔZPE, ΔH, and TΔS were listed at T = 298.15 K, which were obtained from the vibrational calculations of adsorbates (signed by '*') or molecules[15]. The data of ion $Cl^-(g)$ and $H_2O(aq)$ were obtained from the previous reports[16,17].

| **Adsorption species** | **△ZPE** | **△H** | **T△S** |
|---|---|---|---|
| $HO^*$ | 0.38 | 0.04 | 0.06 |
| $O^*$ | 0.07 | 0.03 | 0.05 |
| $HOO^*$ | 0.47 | 0.06 | 0.10 |
| $Cl^*$ | 0.04 | 0.05 | 0.08 |
| $ClO^*$ | 0.11 | 0.08 | 0.15 |
| $H_2O^*$ | 0.7 | 0.05 | 0.07 |
| $Cl^-(g)$ | 0 | 0.06 | 0.47 |
| $H_2(g)$ | 0.27 | 0.09 | 0.40 |
| $O_2(g)$ | 0.10 | 0.09 | 0.64 |
| $Cl_2(g)$ | 0.03 | 0.10 | 0.69 |
| $H_2O(aq)$ | 0.61 | 0.00 | 0.22 |

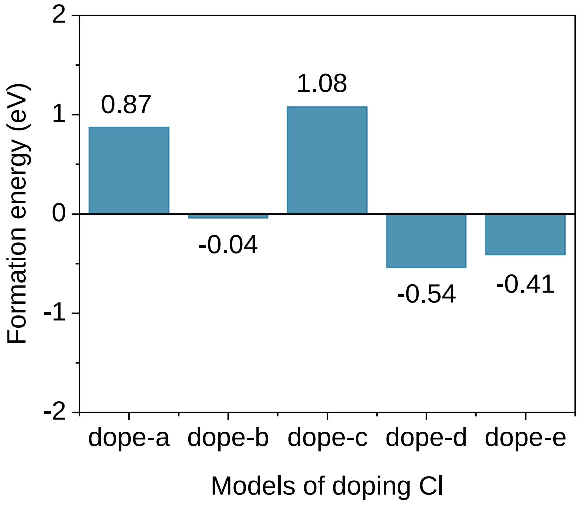


**Supplementary Fig. 48 The formation free energies for the Cl doping in different models.** The negative formation energies suggested that $Cl^-$ was spontaneously introduced into the catalyst. The solvation effect was considered during the calculation.

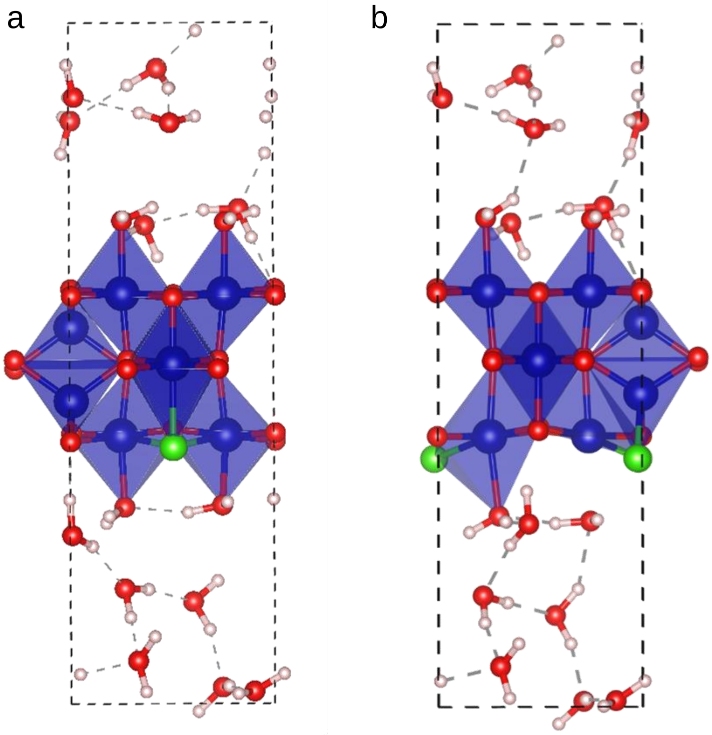


**Supplementary Fig. 49 The optimized two structure models with the lowest formation energy were used for DFT calculation. (a)** The calculation model with Cl doping in the d site. **(b)** The calculation model with Cl doping in the e site. The blue, red, green, and white balls represent the cobalt, oxygen, chlorine, and hydrogen atom, respectively.

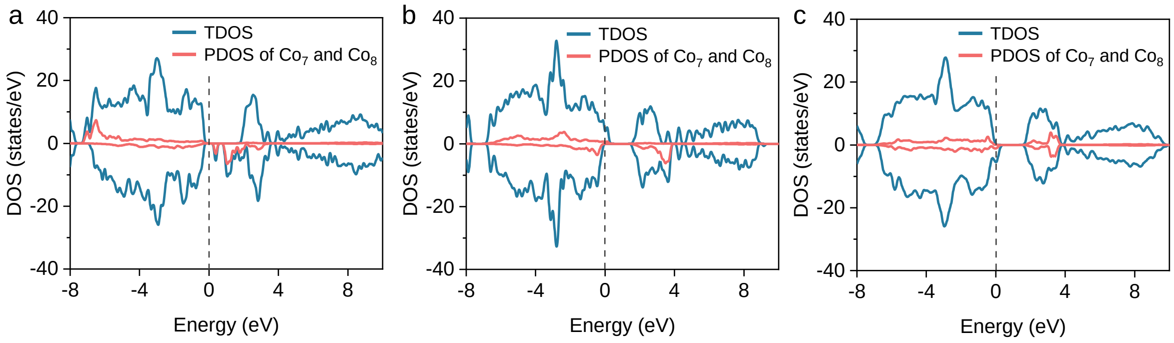


**Supplementary Fig. 50 The density of states.** The total density of states (TDOS) and projected density of states (PDOS) of **(a)** Cl-free model, **(b)** Cl doping in the d site, and **(c)** Cl doping in the e site. Here $Co_7$ and $Co_8$ are located in tetrahedral. The dash line indicates Fermi level.

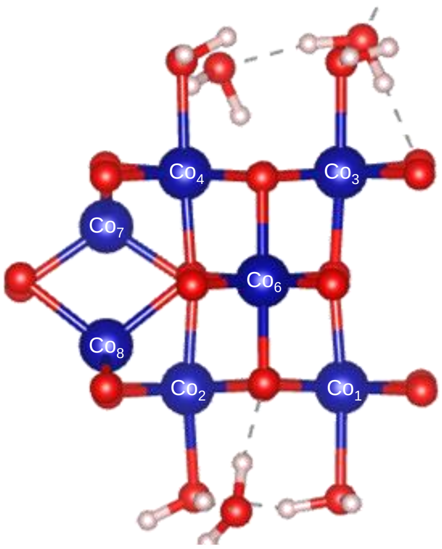


**Supplementary Fig. 51 The optimized calculation models without Cl doping.** The blue, red, and white balls represent the cobalt, oxygen, and hydrogen atom, respectively.

**Supplementary Table 7 The Bader charge on Co sites for the model without Cl doping.** The surface oxygen is coordinated with only three Co atoms, as opposed to the internal oxygen with four Co atoms, which decreases the Bader charges and the bond lengths of $Co_7$ and $Co_8$ in the tetrahedron and implies the oxidation state increase from +2 to +4 according to Supplementary Table 2.

| **Model systems** | **$H_2O^*$ (16 $H_2O$)** | | |
|---|---|---|---|
| **without doping** | Charge | Magnetic moment | Bond length |
| $Co_1$ | 7.65 | 0.10 | 1.94 |
| $Co_2$ | 7.65 | 0.10 | 1.94 |
| $Co_3$ | 7.65 | 0.07 | 1.95 |
| $Co_4$ | 7.67 | 0.07 | 1.95 |
| $Co_5$ | 7.67 | 0.08 | 1.93 |
| $Co_6$ | 7.67 | 0.07 | 1.94 |
| $Co_7$ | 7.54 | 2.95 | 1.90 |
| $Co_8$ | 7.52 | 2.97 | 1.91 |

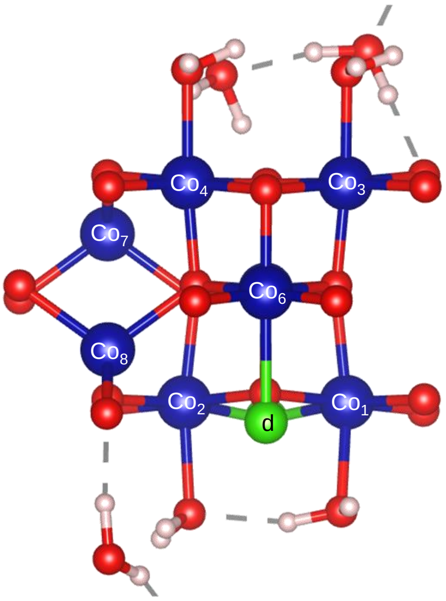


**Supplementary Fig. 52 The optimized calculation models with Cl doping in the d site.** The blue, red, green, and white balls represent the cobalt, oxygen, chlorine, and hydrogen atom, respectively.

**Supplementary Table 8 The Bader charge on Co sites for the model with Cl doping in the d site.** When the surface oxygen is replaced with Cl atom, the Bader charges and the bond lengths of $Co_1$, $Co_2$, $Co_7$, and $Co_8$ are increased compared with Supplementary Table 4, which implies the oxidation states decrease according to Supplementary Table 2.

| **Model systems** | **$H_2O^*$ (16 $H_2O$)** | | |
|---|---|---|---|
| **Cl-doped-d** | Charge | Magnetic moment | Bond length |
| $Co_1$ | 7.71 | 0.14 | 2.00 |
| $Co_2$ | 7.70 | 0.09 | 2.00 |
| $Co_3$ | 7.64 | 0.02 | 1.93 |
| $Co_4$ | 7.66 | 0.03 | 1.94 |
| $Co_5$ | 7.66 | 0.03 | 1.93 |
| $Co_6$ | 7.69 | 0.04 | 1.99 |
| $Co_7$ | 7.70 | 2.61 | 1.94 |
| $Co_8$ | 7.71 | 2.77 | 1.96 |

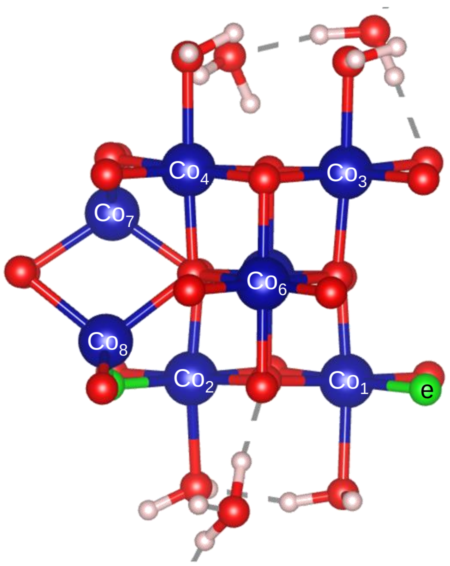


**Supplementary Fig. 53 The optimized calculation model with Cl doping in e site.** The blue, red, green, and white balls represent the cobalt, oxygen, chlorine, and hydrogen atom, respectively.

**Supplementary Table 9 The Bader charge on Co sites for the model with Cl doping in e site.** When the surface oxygen is replaced with Cl atom, the Bader charges and the bond lengths of $Co_1$, $Co_2$, $Co_7$, and $Co_8$ are increased compared with Supplementary Table 4, which implies the oxidation states decrease according to Supplementary Table 2.

| **Model systems** | **$H_2O^*$ (16 $H_2O$)** | | |
|---|---|---|---|
| **Cl-doped-e** | Charge | Magnetic moment | Bond length |
| $Co_1$ | 7.74 | 0.02 | 2.00 |
| $Co_2$ | 7.72 | 0.02 | 1.99 |
| $Co_3$ | 7.68 | 0.11 | 1.93 |
| $Co_4$ | 7.66 | 0.12 | 1.94 |
| $Co_5$ | 7.66 | 0.02 | 1.93 |
| $Co_6$ | 7.66 | 0.01 | 1.94 |
| $Co_7$ | 7.71 | -2.48 | 1.94 |
| $Co_8$ | 7.76 | 2.65 | 2.00 |

**Supplementary Note 5 OER and CER reaction free energies of model systems.**

The surface phase diagram for an electrochemical system as a function of electrode potential (U) and pH provides thermodynamically stable adsorbate structures. In our work, we construct the surface phase diagrams for the model structures both without Cl doping and with Cl doping in the d and e sites for all the plausible adsorbates (Cl*, ClO*, $H_2O$*, HO*, O*, and HOO*). And the * represents the adsorbed states on the surface.

(1) The involved reactions for constructing the OER reaction free energies diagrams :

$H_2O^* \rightarrow HO^* + H^+ + e^-$ (1a)

$H_2O^* \rightarrow O^* + 2H^+ + 2e^-$ (1b)

$H_2O^* + H_2O(aq) \rightarrow HOO^* + 3H^+ + 3e^-$ (1c)

Accordingly, the free energy is calculated as：

dG(HO*) (U, pH) = G(HO*) + 1/2G($H_2$) – G($H_2O$*) – e × (0.0591 × pH + U(vs. SHE))

= dG(HO*) – e × (0.0591 × pH + U(vs. SHE)) = dG(HO*) – e × U(vs.RHE) (2a)

dG(O*) (U, pH) = G(O*) + G($H_2$) – G($H_2O$*) – 2e × (0.0591 × pH + U(vs. SHE))

= dG(O*) – 2e × (0.0591 × pH + U(vs. SHE)) = dG(O*) – 2e × U(vs.RHE) (2b)

dG(HOO*) (U, pH) = G(HOO*) + 3/2G($H_2$) – G($H_2O$*) – G($H_2O$(aq)) - 3e × (0.0591 × pH + U(vs. SHE)) = dG(HOO*) – 3e × (0.0591 × pH + U(vs. SHE)) = dG(HOO*) – 3e × U(vs.RHE)

(2c)

(2) The involved reactions for constructing the CER reaction free energies diagrams:

$H_2O^* + Cl^- \rightarrow Cl^* + H_2O\ (aq) + e^-$ (3a)

$H_2O^* + Cl^- \rightarrow ClO^* + 2H^+ + 3e^-$ (3b)

Accordingly, the free energy is calculated as：

dG(Cl*) (U, pH, α($Cl^-$)) = G(Cl*) + G($H_2O_{(aq)}$) – G($H_2O$*) – G($Cl^-$) – e × (0.0591 × log(α($Cl^-$)) + U(vs. SHE)) = dG(Cl*) – e × (0.0591 × log(α($Cl^-$)) + U(vs. RHE) – 0.0591 × pH) (4a)

dG(ClO*) (U, pH, α($Cl^-$)) = G(ClO*) + G($H_2$) – G($H_2O$*) – G($Cl^-$) – e × (0.1182 × pH + 0.0591 × log(α($Cl^-$)) + 3U(vs. SHE)) = dG(ClO*) – e × (0.1182 × pH + 0.0591 × log(α($Cl^-$)) + 3U(vs. SHE)) = dG(ClO*) – e × (0.0591 × log(α($Cl^-$)) + 3U(vs. RHE) – 0.0591 × pH) (4b)

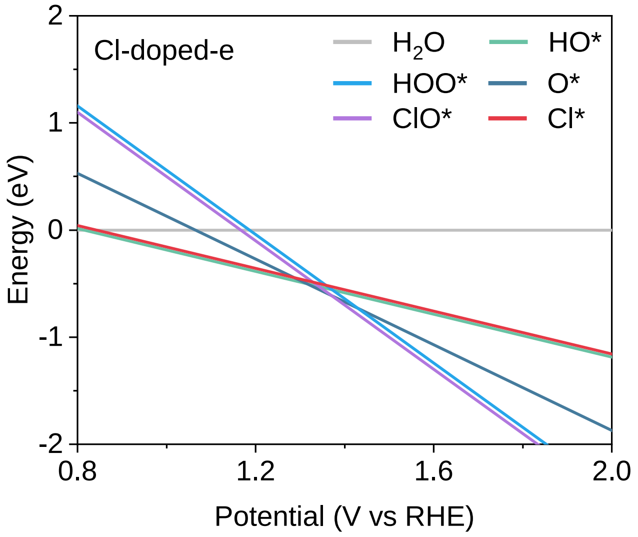


**Supplementary Fig. 54 The surface phase diagram.** The surface phase diagram of the Cl doping in the e site was constructed under different potentials.

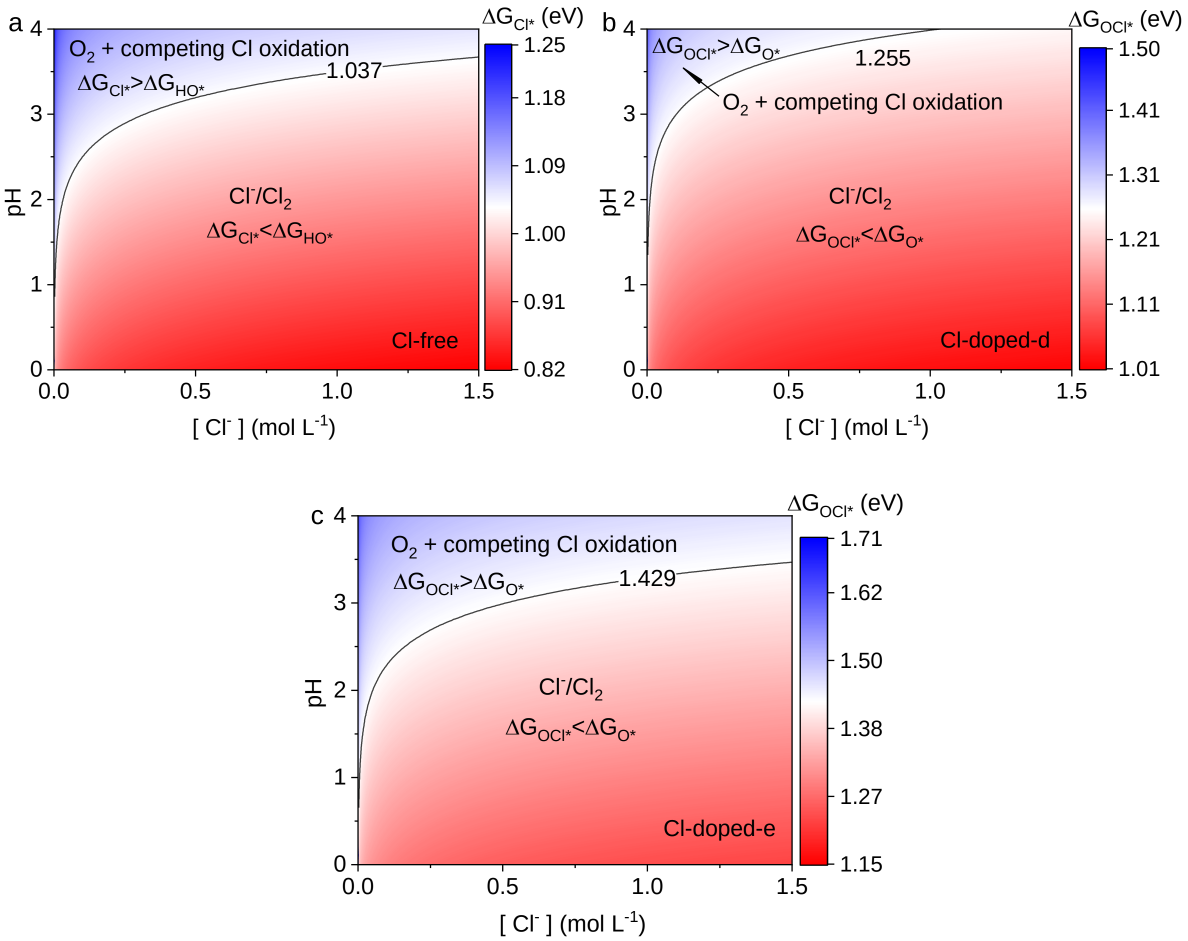


**Supplementary Fig. 55 The calculated reaction free energy diagram under different $Cl^-$ concentrations and pHs based on Supplementary Note 5.** The calculated reaction free energy $\Delta G(Cl^*)$ for **(a)** the model without Cl doping, $\Delta G(OCl^*)$ for **(b)** the model with Cl doping in the d site, and **(c)** the model with Cl doping in the e site. The contour line with 1.037 eV in (a) indicates the reaction free energy of OH* ($\Delta G_{OH*}$). The contour lines with 1.255 and 1.429 eV indicate the reaction free energy of O* ($\Delta G_{O*}$) in (b) and (c), respectively.

**Supplementary Note 6 Mechanism of CER and OER.**

To theoretically evaluate the CER selectivity, we calculate the free energy diagrams for both the structure models without Cl doping and with Cl doping in the d and e sites. The anodic CER usually follows the scheme developed by the Volmer-Heyvrosky reaction[18,19]. The CER is assumed to contain two elementary reaction steps and each step involves electron transfer as follows:

$$Cl^- (g) + * \rightarrow Cl^* + e^- \qquad (5a)$$

$$Cl^* + Cl^- (g) \rightarrow * + Cl_2 (g) + e^- \qquad (5b)$$

Here, * is an active site, which may be surface oxygen or a metal atom.

Hence, two possible reaction pathways involving different reaction intermediates are considered, which are described as follows:

Pathway 1mediated by Cl* intermediate (CER1):

$$* + Cl^- (g) \rightarrow Cl^* + e^- \qquad (6a)$$

$$Cl^* + Cl^- (g) \rightarrow Cl_2 (g) + e^- \qquad (6b)$$

Pathway 2 mediated by ClO* intermediate (CER2):

$$O^* + Cl^- (g) \rightarrow ClO^* + e^- \qquad (7a)$$

$$ClO^* + Cl^- (g) \rightarrow Cl_2 (g) + O^* + e^- \qquad (7b)$$

Accordingly, the reaction free energy for CER can be calculated as follow (U(vs. RHE) = 0):

$$\Delta G(Cl^*) (pH, \alpha(Cl^-)) = dG(Cl^*) – e \times 0.0591 \times \log(\alpha(Cl^-)) + 0.0591 \times e \times pH \qquad (6c)$$

$$\Delta G(ClO^*) (pH, \alpha(Cl^-)) = dG(ClO^*) – dG(O^*) - e \times 0.0591 \times \log(\alpha(Cl^-)) + 0.0591 \times e \times pH \ (7c)$$

The thermodynamic overpotential for CER ($\eta_{CER}$) can be defined as follows:

For the Cl* species

$$\eta_{CER} = | \Delta G (Cl^*) (pH, \alpha(Cl^-)) / e – 1.36 V | \qquad (8)$$

For the ClO* species

$$\eta_{CER} = | \Delta G (ClO^*) (pH, \alpha(Cl^-)) -/ e – 1.36 V | \qquad (9)$$

Where the $\alpha(Cl^-)$ is the concentration of $Cl^-$ in the electrolyte.

The oxygen evolution reaction (OER) usually follows the scheme developed by Nørskov et al[20,21]. The OER is assumed to contain four elementary reaction steps and each step involves electron transfer accompanied by proton expulsion as follows:

$$H_2O\ (aq) + * \rightarrow HO* + H^+ + e^- \quad (10a)$$

$$HO* \rightarrow O* + H^+ + e^- \quad (10b)$$

$$O* + H_2O\ (aq) \rightarrow HOO* + H^+ + e^- \quad (10c)$$

$$HOO* \rightarrow * + O_2\ (g) + H^+ + e^- \quad (10d)$$

Where the symbol * denotes the active site. Accordingly, we can get the reaction free energy formula for the intermediate states (U (vs.RHE) = 0):

$$\Delta G_1 = dG(HO*)\ (U, pH) = dG(HO*) = G(HO*) + G(H^+) + \mu(e^-) – G(*) – G(H_2O) \quad (11a)$$

$$\Delta G_2 = dG(O*)\ (U, pH) – dG(HO*)\ (U, pH) = dG(O*) – dG(HO*) = G(O*) + 2G(H^+) + 2\mu(e^-) – G(*) – G(H_2O) \quad (11b)$$

$$\Delta G_3 = dG(HOO*)\ (U, pH) – dG(O*)\ (U, pH) = dG(HOO*) – dG(O*) = G(HOO*) + 3G(H^+) + 3\mu(e^-) – G(*) - 2G(H_2O) \quad (11c)$$

$$\Delta G_4 = dG(* + O_2) = G(O_2) + 4G(H^+) + 4\mu(e^-) - 2G(H_2O) = 4.92 – (dG_1 + dG_2 + dG_3) \quad (11d)$$

The thermodynamic overpotential for OER ($\eta_{OER}$) can be defined as follows:

$$\eta_{OER} = \max\ (\Delta G_1, \Delta G_2, \Delta G_3, \Delta G_4) / e – 1.23V \quad (12)$$

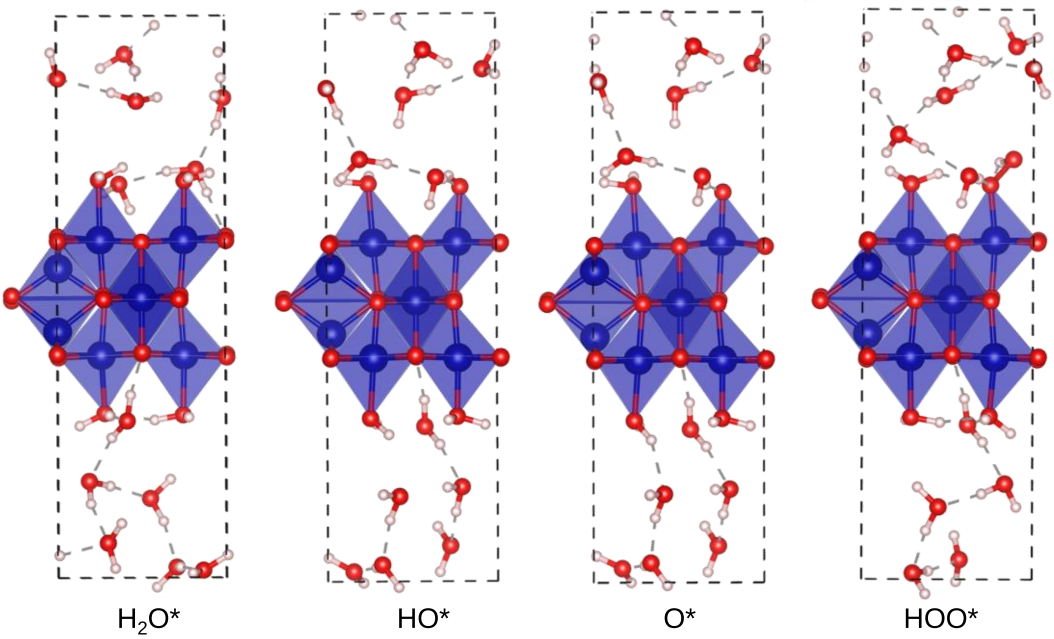


**Supplementary Fig. 56 The calculation models for OER without Cl doping.** The blue, red, and white balls represent the cobalt, oxygen, and hydrogen atom, respectively.

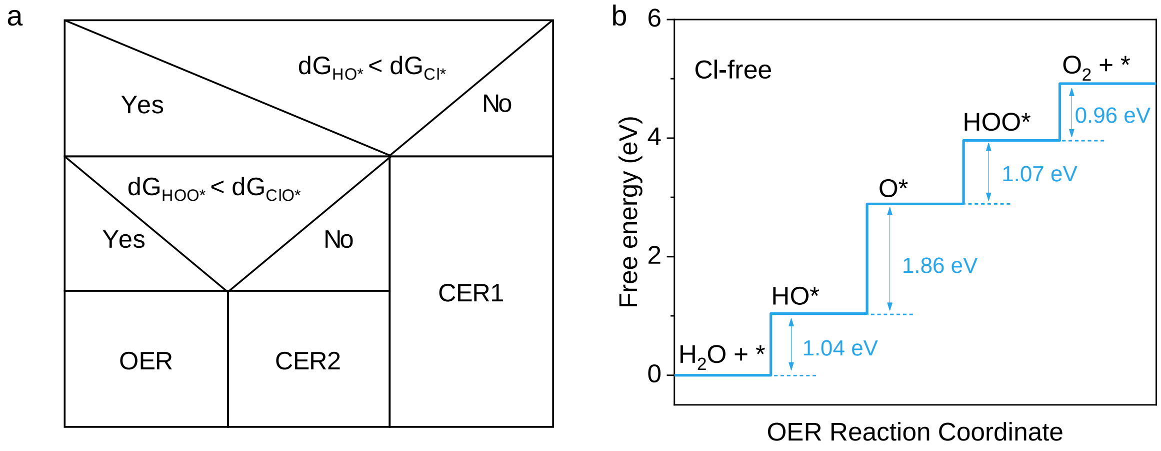


**Supplementary Fig. 57 The reaction free energy diagrams for the calculation models without Cl doping. (a)** The criteria for judging the OER and CER reaction pathways. The CER1 and CER2 see Supplementary Note 6. **(b)** The reaction free energy diagram for OER on the model without Cl doping.

**Supplementary Table 10 The reaction free energies for OER on the Cl-free model.**

| Model systems | dG(HO$^*$) | dG(O$^*$) | dG(HOO$^*$) | dG($^*$) | dG1 | dG2 | dG3 | dG4 |
|---|---|---|---|---|---|---|---|---|
| Cl-free | 1.037 | 2.892 | 3.962 | 4.920 | 1.037 | 1.855 | 1.070 | 0.958 |

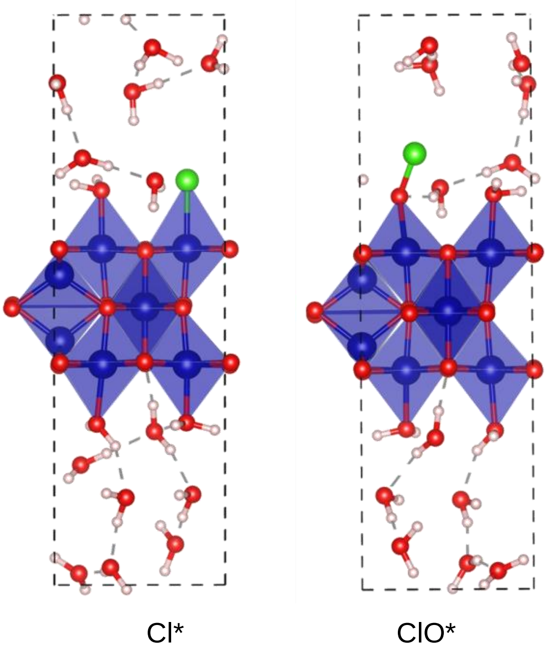


**Supplementary Fig. 58 The calculation models for CER on the Cl-free models. The blue, red,** green, and white balls represent the cobalt, oxygen, chlorine, and hydrogen atom, respectively.

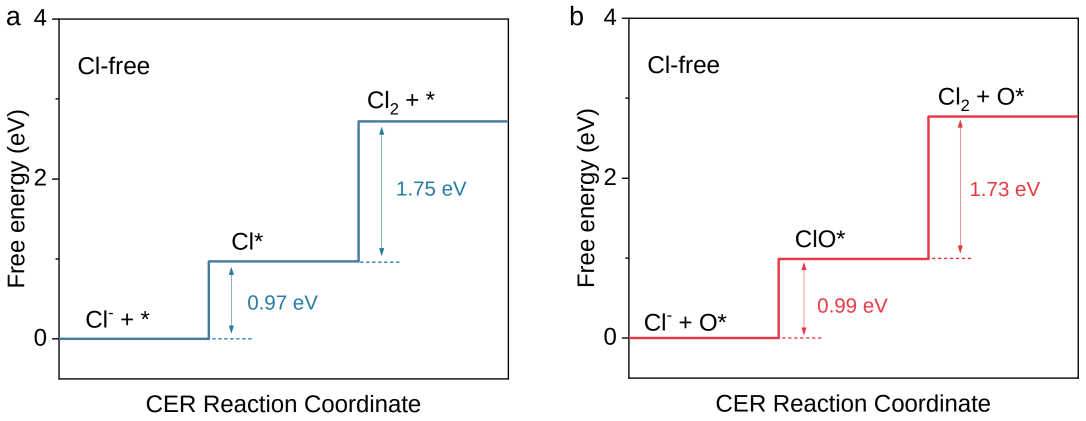


**Supplementary Fig. 59 The reaction free energy diagrams for CER on the calculation models without Cl doping. (a)** The reaction free energy diagrams for pathway 1 are mediated by Cl* intermediate (CER1). **(b)** The reaction free energy diagrams for pathway 2 are mediated by ClO* intermediate (CER2).

**Supplementary Table 11 The reaction free energies for CER on the Cl-free model.**

| Model systems | pH | α ($Cl^-$) | dG($Cl^*$) | dG($ClO^*$) | Paths | Vop |
|---|---|---|---|---|---|---|
| Cl-free | 2 | 0.5 | 0.967 | 3.877 | CER1 | 1..753 |

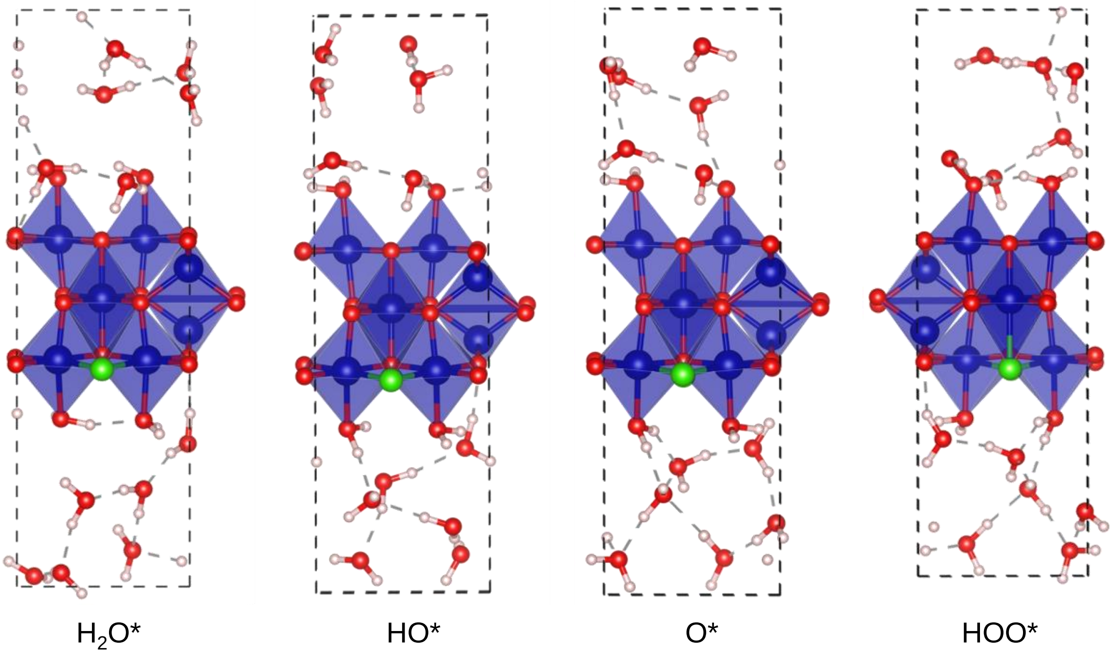


**Supplementary Fig. 60 The calculation models for OER with Cl doping in the d site.** The blue, red, green, and white balls represent the cobalt, oxygen, chlorine, and hydrogen atom, respectively.

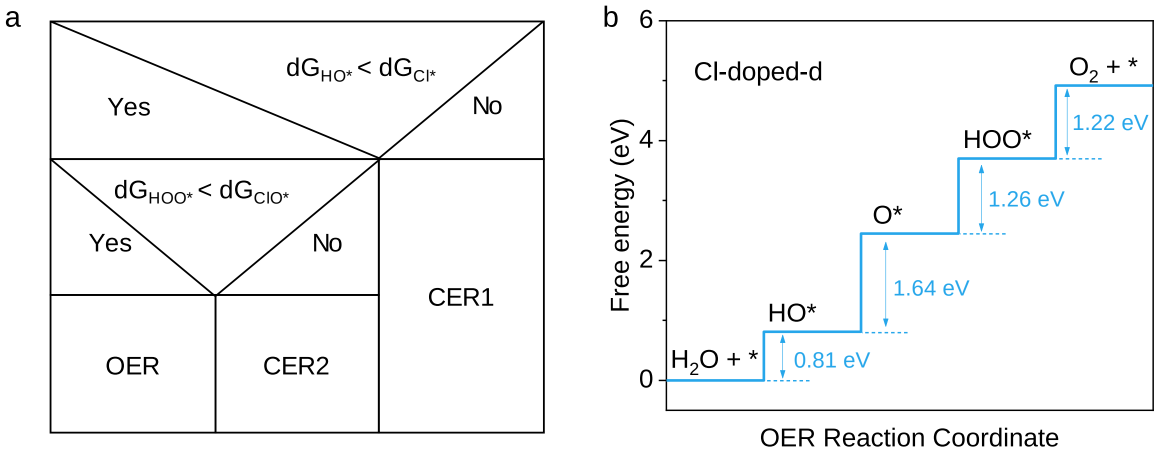


**Supplementary Fig. 61 The reaction free energy diagrams for calculation models with Cl doping in the d site. (a)** The criteria for judging the OER and CER reaction pathways. The CER1 and CER2 see Supplementary Note 6. **(b)** The reaction free energy diagram for OER on the Co site of the Cl-doped model.

**Supplementary Table 12 The reaction free energies for OER on the Cl-doped model.**

| Model systems | dG(HO$^*$) | dG(O$^*$) | dG(HOO$^*$) | dG($^*$) | dG1 | dG2 | dG3 | dG4 |
|---|---|---|---|---|---|---|---|---|
| Cl-doped-d | 0.810 | 2.448 | 3.703 | 4.920 | 0.810 | 1.638 | 1.255 | 1.217 |

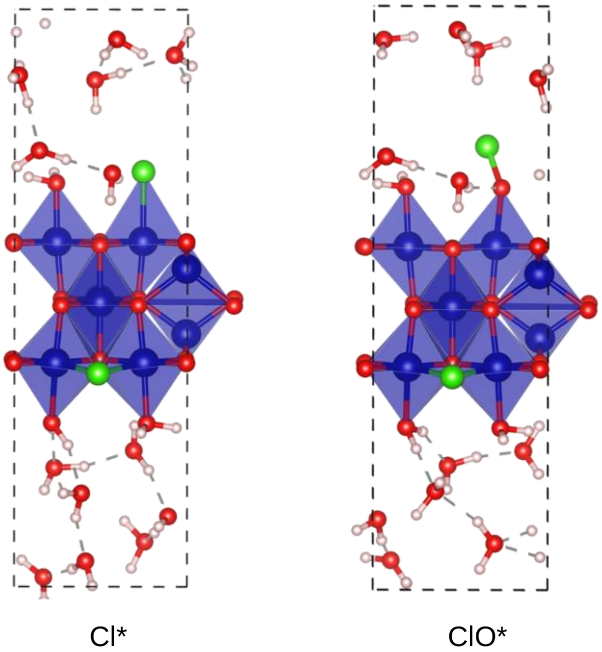


**Supplementary Fig. 62 The calculation models for CER with Cl doping in the d site.** The blue, red, green, and white balls represent the cobalt, oxygen, chlorine, and hydrogen atom, respectively.

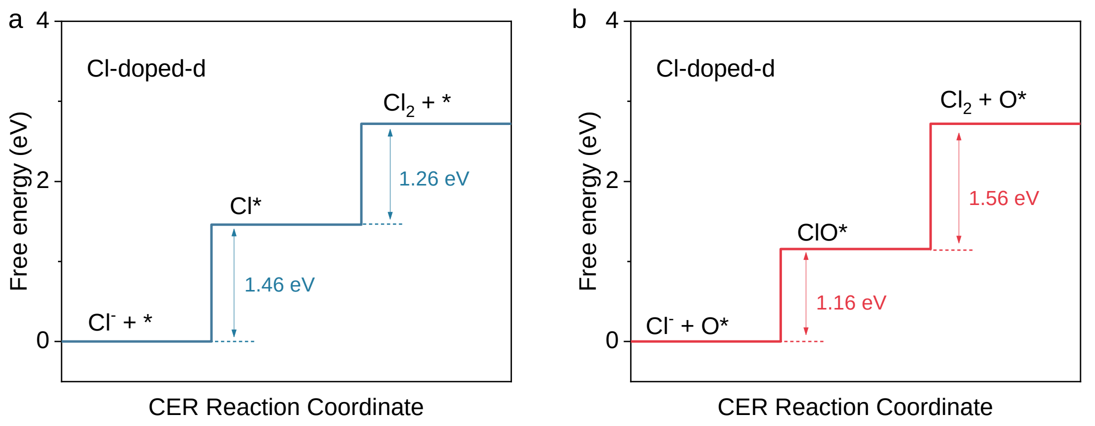


**Supplementary Fig. 63 The reaction free energy diagrams for CER on the models with Cl doping in the d site. (a)** The reaction free energy diagrams for pathway 1 are mediated by Cl* intermediate (CER1). **(b)** The reaction free energy diagrams for pathway 2 are mediated by ClO* intermediate (CER2).

**Supplementary Table 13 The reaction free energies for CER on the model with Cl doping in the d site.**

| Model systems | pH | α($Cl^-$) | dG($Cl^*$) | dG($ClO^*$) | Paths | Vop |
|---|---|---|---|---|---|---|
| Cl-doped-d | 2 | 0.5 | 1.464 | 3.604 | CER2 | 1.638 |

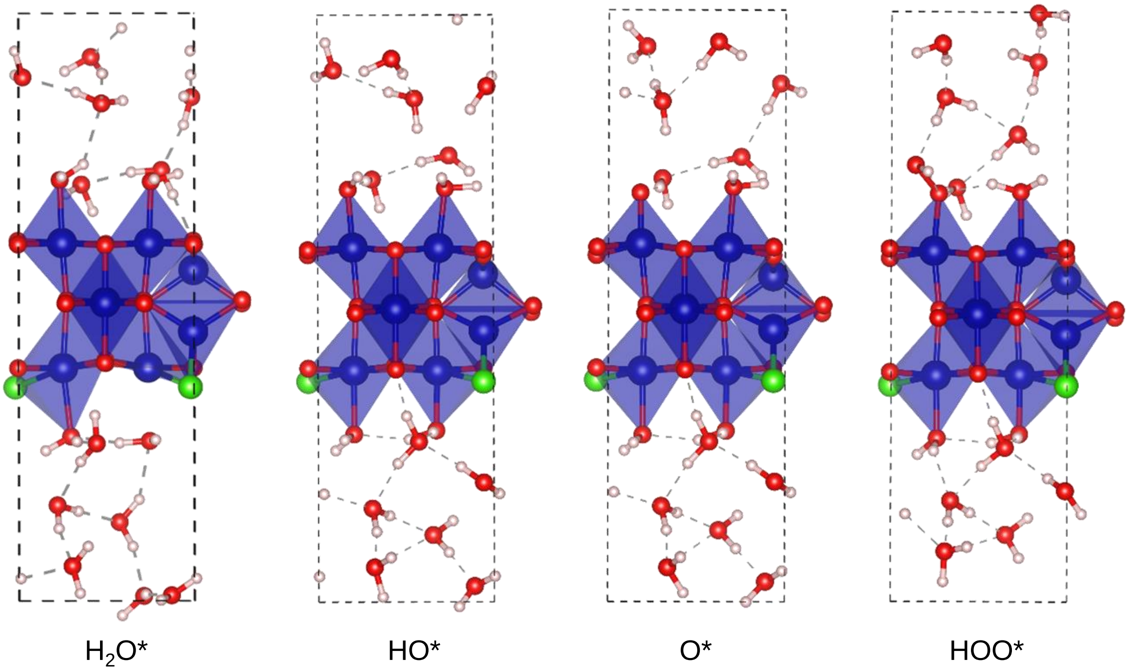


**Supplementary Fig. 64 The calculation models for OER with Cl doping in e site.** The blue, red, green, and white balls represent the cobalt, oxygen, chlorine, and hydrogen atom, respectively.

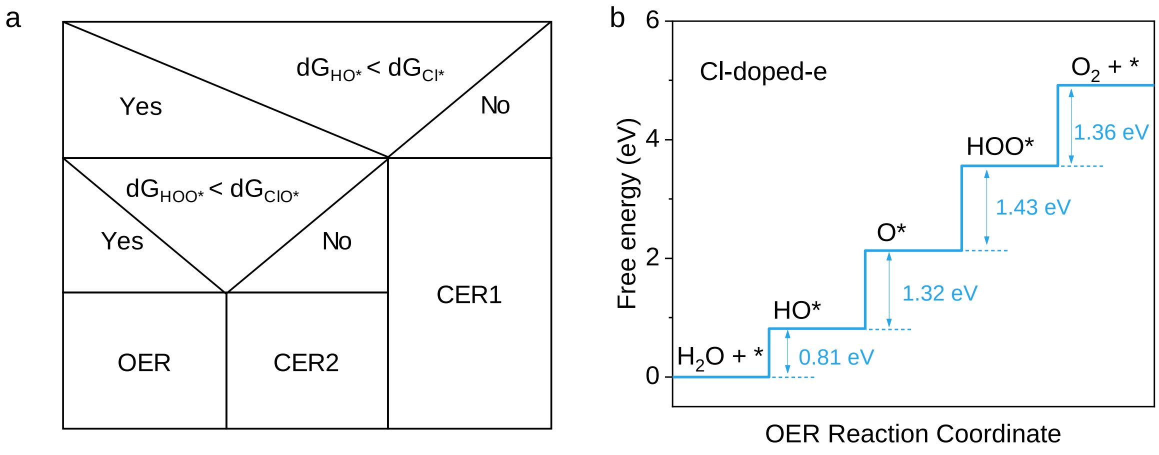


**Supplementary Fig. 65 The reaction free energy diagrams for the calculation models with Cl doping in e site. (a)** The criteria for judging the reaction pathways. The CER1 and CER2 see Supplementary Note 6. **(b)** The reaction free energy diagram for OER on the model with Cl doping in the e site.

**Supplementary Table 14 The reaction free energies for OER on the Cl-doped model.**

| Model systems | dG(HO$^*$) | dG(O$^*$) | dG(HOO$^*$) | dG($^*$) | dG1 | dG2 | dG3 | dG4 |
|---|---|---|---|---|---|---|---|---|
| Cl-doped-e | 0.814 | 2.129 | 3.558 | 4.920 | 0.814 | 1.315 | 1.429 | 1.362 |

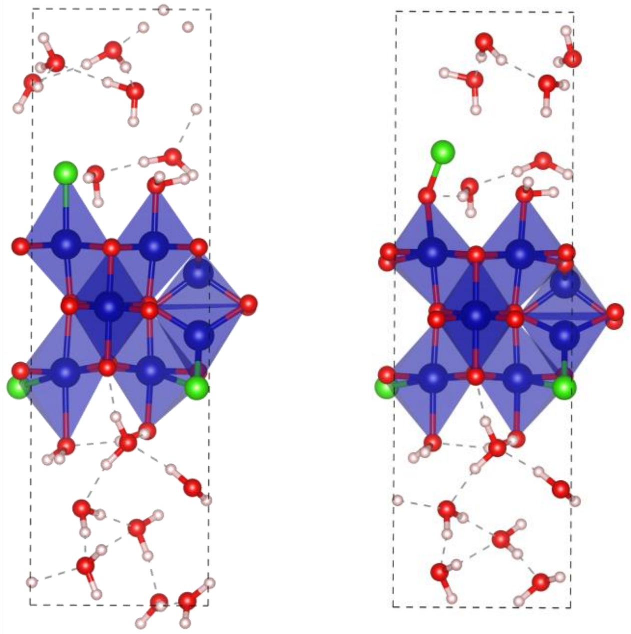


**Supplementary Fig. 66 The calculation models for CER with Cl doping in e site.** The blue, red, green, and white balls represent the cobalt, oxygen, chlorine, and hydrogen atom, respectively.

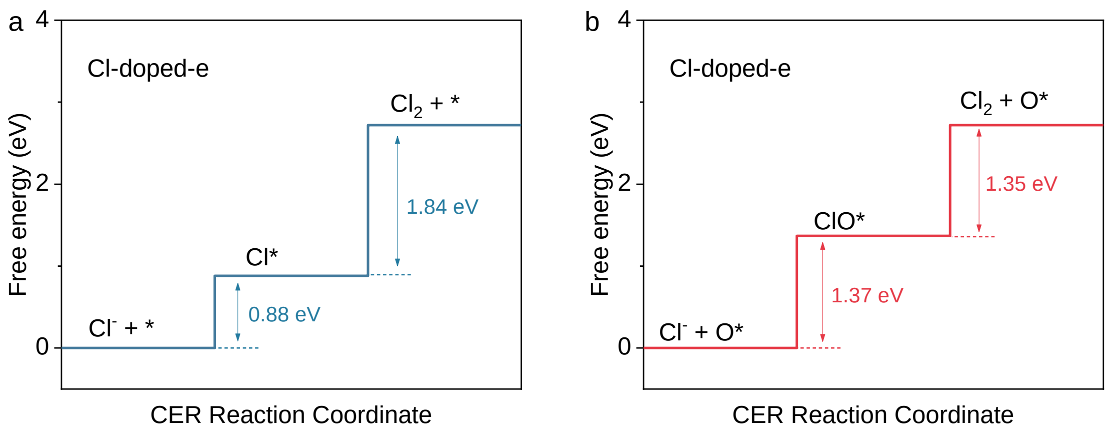


**Supplementary Fig. 67 The reaction free energy diagrams for CER on the calculation models with Cl doping in e site. (a)** The reaction free energy diagrams for pathway 1 are mediated by Cl* intermediate (CER1). **(b)** The reaction free energy diagrams for pathway 2 are mediated by ClO* intermediate (CER2).

**Supplementary Table 15 The reaction free energies for CER on the model with Cl doping in e site.**

| **Model systems** | **pH** | **α(Cl$^-$)** | **dG(Cl$^*$)** | **dG(ClO$^*$)** | **Paths** | **Vop** |
|---|---|---|---|---|---|---|
| Cl-doped-e | 2 | 0.5 | 0.881 | 3.538 | CER2 | 1.371 |

## Supplementary Reference


1 Du, J., Chen, Z., Chen, C. & Meyer, T. J. A half-reaction alternative to water oxidation: Chloride oxidation to chlorine catalyzed by silver ion. *J. Am. Chem. Soc.* **137**, 3193-3196 (2015).

2 Paulus, U. A., Schmidt, T. J., Gasteiger, H. A. & Behm, R. J. Oxygen reduction on a high-surface area Pt/vulcan carbon catalyst: A thin-film rotating ring-disk electrode study. *J. Electroanal. Chem.* **495**, 134-145 (2001).

3 Vos, J. G. et al. Selectivity trends between oxygen evolution and chlorine evolution on iridium-based double perovskites in acidic media. *ACS Catal.* **9**, 8561-8574 (2019).

4 Yin, G. & Zhang, J. Rotating electrode methods and oxygen reduction electrocatalysts. Elsevier (2014).

5 Vos, J. G. & Koper, M. T. M. Measurement of competition between oxygen evolution and chlorine evolution using rotating ring-disk electrode voltammetry. *J. Electroanal. Chem.* **819**, 260-268 (2018).

6 Zhu, X. et al. $Co_3O_4$ nanobelt arrays assembled with ultrathin nanosheets as highly efficient and stable electrocatalysts for the chlorine evolution reaction. *J. Mater. Chem. A* **6**, 12718-12723 (2018).

7 Ha, H. et al. Highly selective active chlorine generation electrocatalyzed by $Co_3O_4$ nanoparticles: Mechanistic investigation through in situ electrokinetic and spectroscopic analyses. *J. Phys. Chem. Lett.* **10**, 1226-1233 (2019).

8 Moreno-Hernandez, I. A., Brunschwig, B. S. & Lewis, N. S. Crystalline nickel, cobalt, and manganese antimonates as electrocatalysts for the chlorine evolution reaction. *Energy Environ. Sci.* **12**, 1241-1248 (2019).

9 Huang, J. et al. $RuO_2$ nanoparticles decorate belt-like anatase $TiO_2$ for highly efficient chlorine evolution. *Electrochim. Acta* **339**, 135878-135886 (2020).

10 Jiang, M. et al. Superaerophobic $RuO_2$-based nanostructured electrode for high-performance chlorine evolution reaction. *Small* **13**, 1602240-1602247 (2017).

11 Deng, L. et al. Preparation of electrolyzed oxidizing water by $TiO_2$ doped $IrO_2$-$Ta_2O_5$ electrode with high selectivity and stability for chlorine evolution. *J. Electroanal. Chem.*

**832**, 459-466 (2019).

12 Zhang, Q. Y., He, X. & Zhao, L. Macrocycle-assisted synthesis of non-stoichiometric silver(i) halide electrocatalysts for efficient chlorine evolution reaction. *Chem. Sci.* **8**, 5662-5668 (2017).

13 Lim, T. et al. Atomically dispersed Pt-N4 sites as efficient and selective electrocatalysts for the chlorine evolution reaction. *Nat. Commun.* **11**, 412-422 (2020).

14 Trasatti, S. Progress in the understanding of the mechanism of chlorine evolution at oxide electrodes. *Electrochim. Acta* **32**, 369-382 (1987).

15 Wang, V., Xu, N., Liu, J.-C., Tang, G. & Geng, W.-T. VASPKIT: A user-friendly interface facilitating high-throughput computing and analysis using VASP code. *Comput. Phys. Commun.* **267**, 108033-108051 (2021).

16 Chase, M. W. & Organization, N. I. S. NIST-JANAF thermochemical tables. Vol. 9 Washington, DC: American Chemical Society (1998).

17 Johnson III, R. D. NIST 101. Computational chemistry comparison and benchmark database. (1999).

18 Hansen, H. A. et al. Electrochemical chlorine evolution at rutile oxide (110) surfaces. *Phys. Chem. Chem. Phys.* **12**, 283-290 (2010).

19 Hepel, T., Pollak, F. H. & O'Grady, W. E. Chlorine evolution and reduction processes at oriented single-crystal $RuO_2$ electrodes. *J. Electrochem. Soc.* **133**, 69-75 (2019).

20 Rossmeisl, J., Qu, Z. W., Zhu, H., Kroes, G. J. & Nørskov, J. K. Electrolysis of water on oxide surfaces. *J. Electroanal. Chem.* **607**, 83-89 (2007).

21 Valdes, A., Qu, Z. W., Kroes, G. J., Rossmeisl, J. & Norskov, J. K. Oxidation and photo-oxidation of water on $TiO_2$ surface. *J. Phys. Chem. C* **112**, 9872-9879 (2008).
